# Multimodal imaging and artificial intelligence for response assessment to neoadjuvant therapy in breast cancer: a comprehensive review

**DOI:** 10.1038/s41523-026-00995-0

**Published:** 2026-06-24

**Authors:** Linlin Shao, Mingyu Zhang, Lili Zhang, Fangfang Sun, Hongyu Li, Jiangbo Shao, Yansong Liu, Xinyue Liu, Feng Hu, Lirong Zhao

**Affiliations:** 1 Ultrasound Diagnostic Center, The First Hospital of Jilin University, Changchun, China; 2 Department of Breast Surgery, General surgery center, The First Hospital of Jilin University, Changchun, China

**Keywords:** Cancer, Computational biology and bioinformatics, Medical research, Oncology

## Abstract

This narrative review explores the latest advancements in various imaging modalities combined with artificial intelligence (AI) for monitoring response to neoadjuvant therapy (NT) and predicting pathological complete response (pCR). We provide a detailed analysis of the principles, evaluation metrics, strengths, and limitations of each technique. The integration of multimodal imaging and AI is reshaping the paradigm for evaluating NT efficacy in breast cancer, offering robust support for precision medicine.

## Introduction

Neoadjuvant therapy (NT) refers to a systemic therapeutic strategy administered prior to surgery, primarily encompassing neoadjuvant chemotherapy (NAC), neoadjuvant endocrine therapy, targeted therapy, and neoadjuvant immunotherapy. Currently, NT has become an integral component of the standard comprehensive treatment paradigm for breast cancer (BC)^1^. Among these modalities, NAC represents the most extensively applied and widely utilized therapeutic approach in patients with BC, typically comprising four to eight treatment cycles^2,3^. The overall course of NAC can be broadly delineated into several phases: pretreatment, early treatment, mid-treatment, and post-treatment/pre-surgery. The clinical significance of NT lies not only in rendering initially inoperable patients eligible for surgical intervention and increasing the rate of breast-conserving surgery (BCS), but also in its capacity to effectively eradicate micrometastatic deposits, reduce the extent of surgical resection, diminish surgical complications and the risk of distant recurrence, and provide essential guidance for subsequent postoperative adjuvant therapy.

The ideal objective of NT is the achievement of pathologic complete response (pCR), defined as the absence of residual invasive carcinoma in the resected surgical specimen. Studies have shown that patients who attain pCR exhibit more favorable survival outcomes, and that the prediction of pCR at different phases of treatment may confer distinct clinical benefits^4^. Consequently, pCR has been recommended as a surrogate endpoint for predicting long-term clinical benefits, such as disease-free survival (DFS) and overall survival (OS)^5^. Accurate prediction of pCR facilitates therapeutic efficacy assessment and treatment decision-making^6^, as it may avert overtreatment and mitigate associated toxicities in patients with a poor response, while concurrently enabling timely modifications to the therapeutic regimen^7^.

Given the aforementioned context, the establishment of an accurate, timely, and effective system for evaluating NT efficacy holds substantial clinical significance. An increasing body of research is currently dedicated to predicting pCR through the integration of imaging characteristics with clinical indices, as well as through the dynamic monitoring of imaging alterations before and during NT^8,9^. Accordingly, the present review aims to comprehensively and thoroughly investigate the role and research progress of imaging modalities and artificial intelligence (AI) models in the assessment of NT efficacy. By comparing the diagnostic performance of different imaging methods in predicting pCR and further analyzing their clinical applicability, this review seeks to propose an evidence-based, timepoint-specific imaging evaluation workflow for the assessment of NT efficacy in patients with BC.

## Methods

This review is conducted in the form of a narrative review, aiming to provide a comprehensive overview of the current applications, technological advances, and future directions of imaging and artificial intelligence in the assessment of neoadjuvant therapy response for breast cancer. Although not a systematic review, a structured literature search strategy was employed to ensure systematic coverage of the field and the inclusion of key literature. Relevant English-language articles published between 2020 and 2025, focusing on imaging evaluation and artificial intelligence applications in neoadjuvant therapy for breast cancer, were retrieved primarily from the PubMed and Web of Science databases. The search keywords included “breast cancer” or “breast neoplasm”, “neoadjuvant therapy”, “neoadjuvant chemotherapy”, “imaging”, “ultrasound” or “US”, “magnetic resonance imaging” or “MRI”, “mammography” or “MG”, “positron emission tomography/computed tomography” or “PET/CT”, “emerging tech”, “artificial intelligence”, “machine learning”, “deep learning”, and “radiomics”. During the literature screening process, priority was given to high-quality prospective studies, large-scale retrospective investigations, and published clinical trials. Additionally, select innovative small-scale exploratory studies and animal experiments were included to illustrate advances at the technological frontier.

## Framework for imaging evaluation strategies

**Fig. 1:**
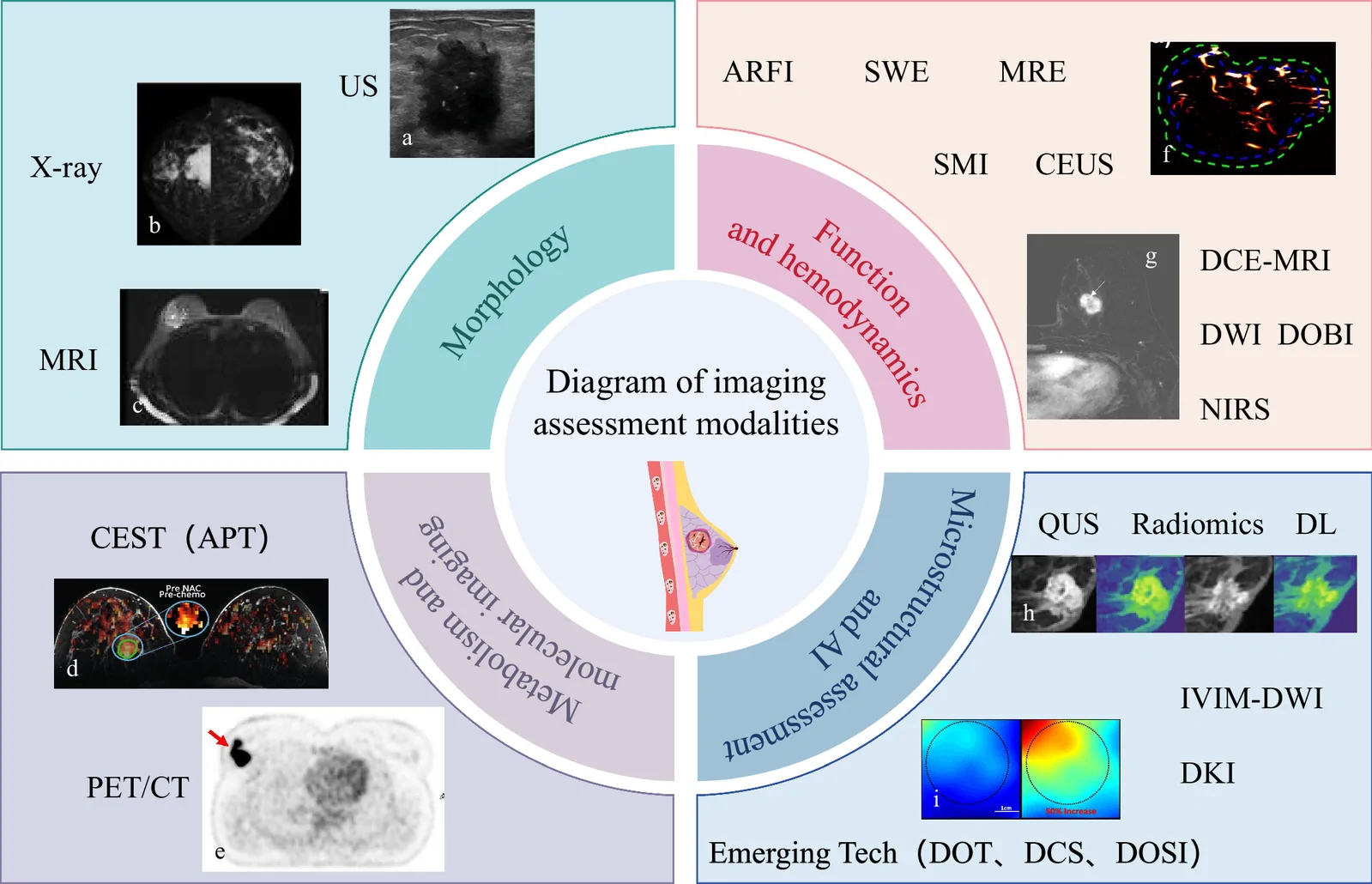
Diagram of imaging assessment modalities. **A** (US) Copyright 2025, Breast Cancer Res Tr, CC BY 4.0^59^; **B** (X-ray) Copyright 2023, Korean J Radiol, CC BY 4.0^71^; **C** (MRI) Copyright 2022; Ann Surg Oncol, CC BY 4.0^111^; **D** (CEST) Copyright 2018, Breast Cancer Res, CC BY 4.0^104^; **E** (PET/CT) Copyright 2024, EJNMMI, CC BY 4.0^126^; **F** (SMI) Copyright 2025, Breast Cancer Res, CC BY 4.0^51^; **G** (DCE-MRI) Copyright 2025, npj Breast Cancer, CC BY 4.0^93^; **H** (BSTNet model) Copyright 2026, Npj Precis Oncol, CC BY 4.0^152^; **I** (DOSI) Copyright 2020, Breast Cancer Res, CC BY 4.0^145^.

Precise imaging evaluation of NT efficacy has evolved from traditional morphometric assessment into a comprehensive predictive framework that closely aligns with the pathological gold standards—pCR and the Residual Cancer Burden (RCB) index^10^. The RCB index is a semiquantitative pathological assessment system that classifies residual tumor burden into grades 0 through III based on the evaluation of residual primary tumor extent, cellular density, and lymph node involvement, wherein RCB-0 corresponds to pCR, and RCB-I, RCB-II, and RCB-III denote mild, moderate, and extensive residual burden, respectively. This index aims to refine prognostic evaluation by analyzing the severity of residual disease following NAC in conjunction with key histopathological features^11^. The objective of imaging is no longer confined to assessing changes in tumor size according to the RECIST 1.1 criteria^12^; rather, it seeks to enable early, non-invasive detection of functional, metabolic, and microenvironmental alterations within the tumor, thereby facilitating early prediction of pathological response and providing a critical basis for the formulation of individualized therapeutic strategies. Accordingly, the contemporary imaging strategy for evaluating NT efficacy can be integrated into a multidimensional, multimodal framework encompassing four core dimensions: morphology, function and hemodynamics, metabolism and molecular imaging, as well as microstructural assessment and AI (A diagram of imaging assessment modalities is presented in Fig. 1).

Conventional imaging modalities such as ultrasonography (US)^13^ and mammography (MG)^14^ primarily provide morphological evaluation, including fundamental anatomical information on tumor location, size, margin characteristics, lymph node morphology, and calcification status, thereby constituting the cornerstone of efficacy assessment.

Functional and hemodynamic imaging techniques have garnered considerable attention in recent years for their capacity to capture functional alterations in tumor stiffness, microcirculatory perfusion, and vascular permeability that precede morphological changes. For instance, ultrasound elastography techniques—including acoustic radiation force impulse (ARFI) imaging and shear wave elastography (SWE)—effectively differentiate fibrosis from viable tumor tissue by quantifying tissue stiffness, measured as shear wave velocity (SWV)^15,16^; similarly, magnetic resonance elastography (MRE) reflects microstructural tissue alterations through mechanical properties, enabling the distinction of active tissue components during efficacy prediction and facilitating functional assessment of tumor dimensions and therapeutic response^17^. Furthermore, superb microvascular imaging (SMI), contrast-enhanced ultrasonography (CEUS), and dynamic contrast-enhanced magnetic resonance imaging (DCE-MRI) enable early assessment of treatment response by monitoring hemodynamic parameters such as perfusion curves, peak intensity, and time to peak^18,19^. Diffusion-weighted imaging (DWI) reflects the degree of restricted water molecule diffusion via the apparent diffusion coefficient (ADC), indirectly indicating treatment-induced reductions in cellular density and necrosis^20^. Optical imaging modalities, including dynamic optical breast imaging (DOBI) and near-infrared spectroscopy (NIRS), assess blood oxygen metabolic function by quantifying hemoglobin concentration and oxygenation status (HbO₂/HHb); these optical techniques have demonstrated promising potential for clinical application^21^.

In the dimension of metabolic and molecular imaging, chemical exchange saturation transfer (CEST) imaging—such as amide proton transfer (APT) imaging—enables non-invasive detection of intracellular protein content and pH alterations^22^; Hyperpolarized ¹³C-MRI permits real-time monitoring of the conversion of key in vivo metabolites (e.g., pyruvate/lactate), thereby directly reflecting glycolytic activity and advancing molecular imaging from macroscopic to microscopic metabolic surveillance^23^. Positron emission tomography/computed tomography (PET/CT) quantifies tumor metabolic activity through the uptake of tracers (e.g., ¹⁸F-FDG) and has become an established and crucial molecular imaging modality for assessing NT efficacy^24,25^.

With ongoing scientific and technological progress, imaging evaluation is increasingly focused on extracting deeper quantitative features to elucidate tumor heterogeneity and microenvironmental characteristics, thereby achieving exceptionally high predictive accuracy. Quantitative ultrasound (QUS) techniques derive acoustic parameters related to tissue microstructure through the analysis of raw radiofrequency signals^26^; radiomics and deep learning (DL) methodologies construct high-precision predictive models by extracting high-throughput handcrafted features and automatically learning patterns via convolutional neural networks (CNNs), respectively; these approaches can further integrate multimodal data, including clinical and genomic information, to enhance model performance^27–29^.

Notably, magnetic resonance imaging (MRI)^30^, as a cross-dimensional imaging modality, integrates three major evaluative dimensions: morphology (T1W/T2W), function (DCE-MRI, DWI, and MRE), and microstructural/omics assessment (intravoxel incoherent motion diffusion-weighted imaging (IVIM-DWI), diffusion kurtosis imaging (DKI), radiomics). Consequently, MRI holds a significant advantage in imaging evaluation and currently represents one of the optimal methods for assessing NT efficacy via imaging. Furthermore, emerging optical technologies such as diffuse optical tomography (DOT)^31^, diffuse correlation spectroscopy (DCS), and diffuse optical spectroscopic imaging (DOSI)^32,33^, which combine functional and microstructural evaluation to provide non-invasive, wide-field hemodynamic and metabolic information, also exhibit considerable developmental promise. These techniques will be discussed in detail in the following sections. Table 1 compares the core evaluation metrics, advantages, and limitations of various imaging techniques.

**Table 1:** Comparative analysis of major imaging modalities for evaluating response to neoadjuvant therapy in breast cancer

Technical category	Representative techniques	Primary evaluation metrics	Advantages	Limitations	Clinical values
US	Conventional US, SWE, SMI, CEUS	Morphology, Stiffness, Blood Flow	Real-time; convenient; no radiation; Low cost; high reproducibility	High operator dependence; Insensitive to small lesions; Potential measurement bias for BC tumor size	Baseline assessment; Therapeutic response assessment
X-ray	MG, DBT, CEM	Morphology, Density, Calcifications	Standardized; Economical; Convenient; High sensitivity for calcifications	Ionizing radiation; Low sensitivity in dense breast tissue	Baseline assessment; Early response assessment; Preoperative assessment
MRI	MRI, DCE-MRI, DWI, MRE, etc.	Morphology, Function, Metabolism, Microstructure	High soft-tissue contrast and spatial resolution	High cost; Limited availability; Long scan time; Metal implant restrictions	Baseline assessment; Therapeutic response assessment; Preoperative assessment
PET/CT	¹⁸F-FDG PET/CT, ⁶⁸Ga-FAPI PET/CT	Metabolic activity	Whole-body imaging; High sensitivity	Lower spatial resolution; Expensive; Radiation; Inflammation can cause false positives	Early response assessment; Preoperative assessment
Emerging Tech	NIRF, DOBI, DOT, MPM, etc.	Oxygen metabolism, Microstructure	Non-irradiating; Provides rich functional information	Shallow tissue penetration; Mostly in preclinical stage	Early response assessment

## Ultrasound, US

Ultrasonography (US), by virtue of its distinct advantages, including the absence of ionizing radiation, operational convenience, and real-time dynamic imaging capability, has become the preferred imaging modality for the evaluation of various diseases affecting the breast^34^, thyroid^35^, and liver^36^. In the context of breast cancer (BC) management, US possesses irreplaceable clinical value in the preoperative staging of axillary lymph nodes (ALN) and the monitoring of neoadjuvant chemotherapy (NAC) efficacy^37^. Its evaluative scope has expanded from conventional morphological assessment to encompass functional and microstructural analyses, thereby constituting a multimodal evaluation system. The specific emphases of different ultrasound techniques in the assessment of neoadjuvant therapy efficacy are summarized in Table 2, along with their core characteristics.

**Table 2:** Comparison of ultrasound techniques for assessing response to neoadjuvant therapy

Technical category	Technique name	Principle	Primary evaluation metrics	Advantages	Limitations	Clinical Application Scenarios	Refs
Morphological assessment	Conventional US	Image generation based on tissue acoustic impedance differences	Tumor maximum diameter; ALN status	Real-time; convenient; no radiation; Low cost; high reproducibility	High operator dependence; Insensitive to small lesions; Potential measurement bias for BC tumor size	Initial morphological evaluation of NT efficacy	^13,42^
	ABUS/ABVS	Reconstruction of breast volume images from 2D US data	Tumor 3D volume	Suitable for dense breast tissue; Cost-effective; Automated; Easy operation	Difficulty distinguishing post-treatment fibrosis from residual cancer; Challenging ALN assessment; Potential tumor size overestimation	Routine follow-up in primary care hospitals	^44^
Functional assessment	ARFI/SWE	Tissue stiffness assessment by measuring shear wave velocity	SWV	Quantitative tissue stiffness; Helps differentiate viable tumor from fibrosis	Measurements affected by necrosis, edema; Depth limitations; Lack of unified diagnostic cut-off values	Early reflection of tumor stiffness changes; Early treatment response monitoring; Supplementary to conventional US to improve specificity	^48^
	SMI/CEUS/CDUS	Hemodynamic monitoring	RI, PSV, TIC, etc.	Assesses tumor microcirculatory perfusion; High resolution; Quantitative analysis	Qualitative analysis is subjective; Limited assessment of diffuse residual foci	Early evaluation of tumor angiogenesis changes; Identifying viable tissue	^18,51^

*ALN* axillary lymph node, *SWV* shear wave velocity, *PSV* peak systolic velocity, *RI* resistance index, *TIC* time-intensity curve; *MD* mammographic density, *TSR* tumor stiffness ratio, *PAR* phase angle ratio, *LAC/PYR* lactate-to-pyruvate ratio, *Hb* hemoglobin, *HbO₂* oxygenated hemoglobin, *BS* blood score, *OS* oxygenation score, *HbT* total hemoglobin concentration, *StO₂* tissue oxygen saturation, *BFI* blood flow index, *MRO₂* metabolic rate of oxygen.

### Conventional US and automated breast ultrasound

As presented in Table 2, conventional US and automated breast ultrasound (ABUS) constitute the foundation of morphological assessment. Research regarding the prediction of pCR based on the intrinsic sonographic features of the mass itself via conventional US remains relatively limited, with relevant predictions largely reliant on radiomics approaches. In contrast, axillary ultrasound (AUS) demonstrates greater predictive value. Studies have indicated that US exhibits favorable overall accuracy (82%) in ALN restaging; however, its predictive value varies according to molecular subtype: a high negative predictive value is observed in the HER2+ subtype, whereas a high positive predictive value is noted in the HER2– subtype^38,39^. Concurrently, this research demonstrates the clinical utility of AUS following NAC (predominantly after the second cycle), particularly in identifying patients likely to achieve axillary pCR, thereby facilitating the selection of suitable candidates for sentinel lymph node biopsy^38^. This discrepancy underscores that clinical decision-making should adhere to individualized principles, wherein sonographic findings must be integrated with molecular subtyping to guide the formulation of lymph node dissection strategies. In the context of TNBC, the detection of more than four abnormal ALNs by AUS at mid-treatment serves as a robust indicator for predicting residual disease (AUC = 0.908; 95% CI: 0.829–0.987)^40^. This finding aids in the early identification of patients with chemoresistance, thereby guiding adjustments to individualized therapeutic regimens and informing axillary surgical planning; nevertheless, the study is constrained by a limited sample size (106 cases) and its single-center design, necessitating further large-scale, multicenter investigations to corroborate the reliability of its findings. Furthermore, Malhaire C et al. were the first to integrate pretreatment MRI features (BI-RADS) with AUS, revealing that tumor depth (anterior one-third region) and lymph node cortical thickness ≥7 mm were also significantly associated with axillary residual disease, a phenomenon potentially attributable to lymphatic drainage pathways^41^. However, this study is similarly limited by a small sample size. Nonetheless, ultrasound assessment possesses inherent limitations. In HER2+ and TNBC subtypes, US tends to overestimate actual tumor dimensions, thereby potentially increasing the risk of unnecessary total mastectomy^42^; conversely, in the ER+/HER2– subtype, influenced by the pattern of “diffuse cell loss,” US may underestimate the true extent of the tumor, thus warranting a cautious approach during surgical planning^43^.

ABUS and the automated breast volume scanner (ABVS) provide standardized three-dimensional assessment modalities, with predictive performance for pCR comparable to that of MRI^44,45^. Radiomics models based on ABVS have demonstrated superior performance in therapeutic response prediction. Jiang W et al. reported that ABVS-based radiomics can effectively predict treatment response in BC patients during the early phase of NAC (after the second cycle). This period constitutes a critical timepoint for efficacy prediction, as the tumor microenvironment has already been influenced by chemotherapy and can more accurately reflect the overall response, thereby assisting clinicians in making early adjustments to the therapeutic regimen^46^. In comparison to advanced imaging examinations, ABUS and ABVS offer advantages such as high cost-effectiveness, ease of operation, and the absence of contrast agent administration, rendering them more amenable to widespread adoption and dissemination in primary healthcare settings. Nevertheless, these modalities exhibit suboptimal sensitivity in distinguishing treatment-induced fibrosis from hypoechoic residual tumor lesions and present certain limitations in the assessment of ALN status.

### Elastography and hemodynamic monitoring

Conventional ultrasonography exhibits inherent limitations in reliably distinguishing viable tumor tissue from fibrotic stroma. Elastography and hemodynamic assessment techniques, capable of capturing functional alterations that precede morphological changes, are therefore pivotal for early therapeutic response prediction. Elastography differentiates active tumor from post-treatment fibrosis by quantifying tissue stiffness, measured as SWV^47^. Multiple studies have confirmed that the rate of decline in SWV or elasticity values during early treatment serves as a robust indicator for predicting pCR^15,16,48^. Similarly, hemodynamic monitoring techniques, such as SMI or CEUS, are employed to probe alterations in tumor microcirculation. Investigations have demonstrated that effective chemotherapy reduces tumor blood supply through anti-angiogenic effects or direct cytotoxicity, and this early decline in blood flow (within 1–2 weeks of treatment initiation) can function as a predictive biomarker of therapeutic response^49^. Using SMI, Qi J et al. observed that the reduction in peak systolic velocity and resistive index was significantly more pronounced in the treatment-sensitive cohort than in the resistant cohort (*p* < 0.05)^50^. The research group led by Sabeti S reported congruent findings, noting characteristic reductions in microvascular density and vascular complexity within 2–4 weeks of treatment in the sensitive group^51^. CEUS not only enables clear visualization of intratumoral microvascular architecture to reflect vessel patency but also permits dynamic and quantitative analysis of tumor perfusion^52^; however, related studies have been relatively sparse in recent years. A more advanced technique, micro-ultrasound localization microscopy (mULM), provides microvascular detail that far surpasses that of conventional CEUS, offering a novel tool for detecting subtle hemodynamic alterations. Rix A et al. explored high-MI CDUS, which may enhance tumor perfusion and drug delivery via the “sonopermeation” effect. Although this approach significantly disrupted microvasculature in animal models without affecting carboplatin distribution—attributable to the fact that high MI disrupts microbubbles, generating transient shock waves that rupture small vessels, whereas the drug predominantly resides in the perivascular space of larger vessels, thus maintaining a constant quantity—it failed to significantly alter tumor perfusion parameters in clinical BC applications. This discrepancy may be ascribed to the higher vascular maturity and abundant stromal composition of human tumors, which mitigate the mechanical effects of US, resulting in insignificant perfusion changes^18^. The aforementioned study innovatively applied mULM to achieve microbubble motion tracking, and the resultant super-resolution vascular network imaging proved significantly superior to conventional CEUS in detecting subtle blood flow variations^18^. These technological advancements indicate that objective quantitative assessment predicated on changes in microvascular parameters not only surmounts the subjective limitations inherent to traditional US but also facilitates early therapeutic response prediction, thereby providing a crucial foundation for the individualized adjustment of treatment strategies.

### Quantitative ultrasound (QUS) and radiomics

QUS quantifies acoustic parameters associated with tissue microstructure through the analysis of raw radiofrequency signals. Research by Karwat et al. demonstrated that a method based on probability maps of QUS parameters can efficiently identify treatment-sensitive regions during mid-chemotherapy (after the fourth cycle). The optimal parameter combination (autocovariance and Laws’ texture energy (SSLS)) achieved a predictive AUC of 0.94 with a sensitivity of 1.00, effectively circumventing the information dilution inherent in whole-tumor averaging^26^. This approach significantly enhanced the identification of non-responders while maintaining a certain specificity, thereby contributing to the avoidance of continued ineffective therapy. Another animal study demonstrated that H-scan US, a novel high-resolution ultrasound imaging technique that characterizes the relative size of tissue scatterers by analyzing frequency shifts in echo signals, may serve as a potential biomarker for monitoring early treatment-induced apoptosis through the detection of reduced scatterer dimensions^53^. Klimonda Z et al. proposed a novel QUS method that analyzes alterations in the amplitude distribution of US backscatter signals. Based on envelope statistics of the US backscatter, the Kolmogorov–Smirnov statistic and symmetric Kullback–Leibler divergence could effectively identify BC patients unresponsive to NAC, providing clinically instructive information as early as the first post-NAC assessment and thus informing individualized treatment regimen adjustments^54^. This technique is independent of tumor size alterations, is non-invasive, and offers repeatable measurements, rendering it suitable for broader application in clinical practice.

With innovations in medical imaging technology and breakthroughs in AI algorithms, image analysis methods based on radiomics and machine learning (ML) are progressively transforming the clinical practice of evaluating BC NAC efficacy. As core branches within the AI domain, radiomics and DL enable the quantitative analysis of high-dimensional features extracted from medical images such as US, thereby capturing microscopic pathological alterations that are often imperceptible to conventional approaches. Currently, such methodologies have demonstrated significant potential in the imaging assessment of various organs, including the thyroid^55^, heart^56^, and liver^57^.

Early models, through the extraction of intratumoral textural features, have exhibited relatively stable performance in predicting ALN status, with AUC values ranging from 0.855 to 0.882^58^. Regarding the assessment of peritumoral characteristics for predicting pCR, Yao J et al. conducted a systematic comparison of intratumoral and 3-mm peritumoral regions prior to treatment. They discovered that an intratumoral model constructed using the random forest algorithm demonstrated particularly prominent efficacy in predicting pCR (test set AUC = 0.931; 95% CI: 0.865–0.980), outperforming the peritumoral model^59^. A similar conclusion was reached in another study by ref. ^60^. Nevertheless, that investigation arbitrarily fixed the peritumoral region at 3 mm and did not explore whether other expanded distances might yield superior predictive performance; consequently, the robustness of its findings warrants further validation. A retrospective study by Wang M et al. involving 402 BC patients elucidated the influence of the peritumoral region on predictive performance. By analyzing pretreatment patient images, the study revealed that as the peritumoral margin expanded, the performance of the DL model exhibited an initial increase followed by a decline, with the model incorporating a 6-mm region (R6) performing optimally (AUC = 0.845). It is also noteworthy that although the R6 model achieved high accuracy (81.0%) and precision (76.5%), its comparatively lower recall rate (63.4%) suggests that this model is more suitably applied for ruling out non-responders to therapy. The study further integrated intratumoral features with those from the 6-mm peritumoral region to construct a combined model (R0 + R6), which resulted in a substantial enhancement of predictive performance, achieving an accuracy of 90.9%, precision of 84.1%, recall of 90.2%, and an F1 score of 0.871^61^. This performance improvement may be attributable to the 6-mm peritumoral region precisely encompassing the critical biological territory of tumor-stroma interaction (e.g., immune cell infiltration and vascular remodeling), whereas signals from more distant regions may introduce greater noise. This investigation is the first to systematically establish 6 mm as the optimal threshold for peritumoral radiomics analysis, thereby providing a reference for clinical practice. However, the study excluded patients with multifocal BC, excessively large tumors, or superficially located lesions; therefore, the generalizability of its model requires further investigation.

**Fig. 2:**
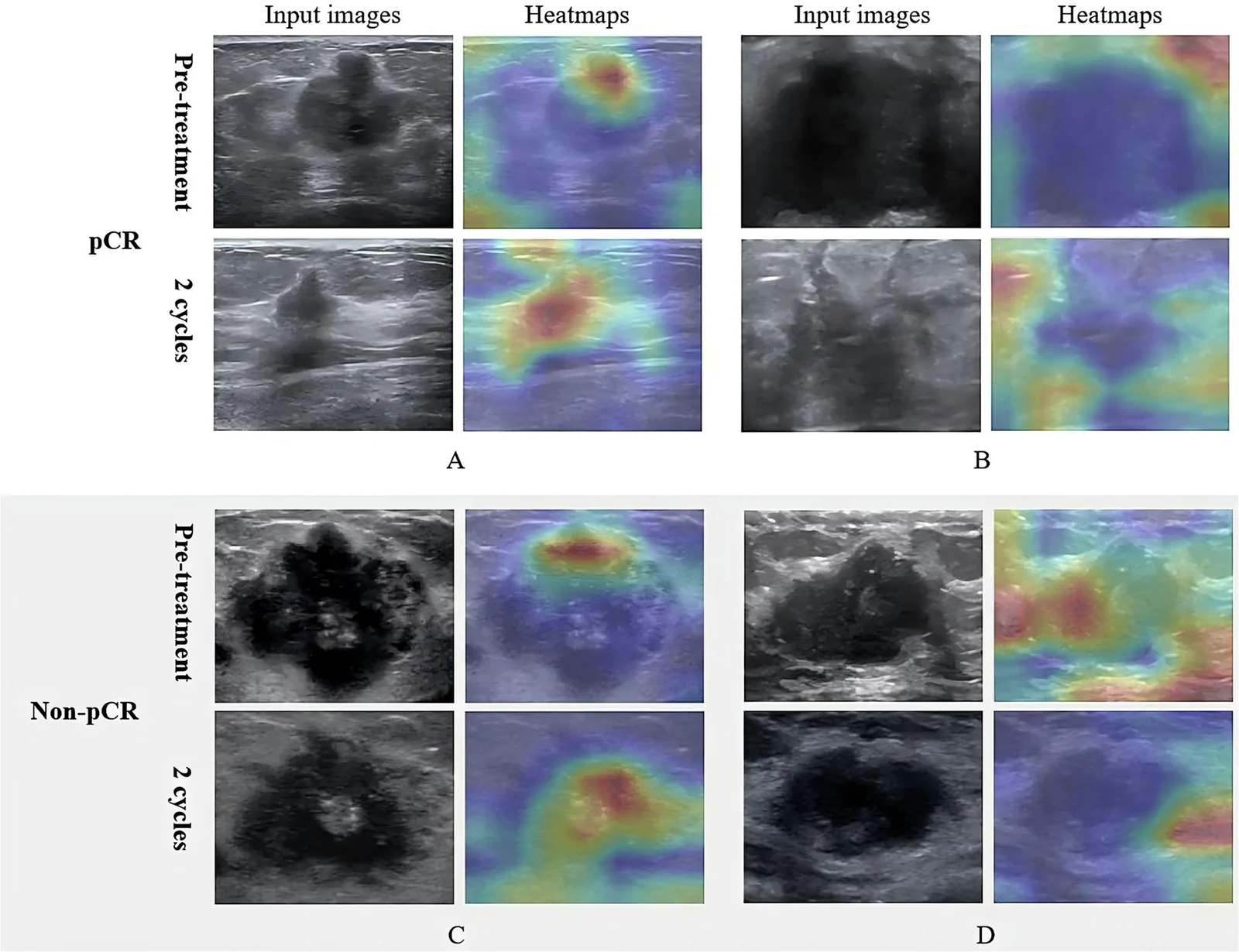
Example of Grad-CAM visualization for the model. Grad-CAM visualization provides four predictions for the model: true positives (**A**), true negatives (**C**), false positives (**D**), and false negatives (**B**). The red and yellow regions represent areas of model activation with the greatest predictive significance for the results, whereas the green and blue regions reflect areas of weaker predictive significance. Copyright 2025, Breast Cancer Res, CC BY 4.0.

The research frontier is shifting from static analysis toward dynamic monitoring and multimodal fusion. Feng X et al. constructed a multi-timepoint DL model, confirming that feature alterations during the course of treatment possess greater predictive value than baseline characteristics (A schematic diagram of the model predicting pCR versus non-pCR is shown in Fig. 2)^62^. Guo J et al. innovatively integrated whole-slide pathological images with multi-timepoint US images(pre-NAC and after the second cycle). Their cross-modal model outperformed unimodal approaches (AUC = 0.875; 95% CI: 0.835–0.890)^63^. Furthermore, the model developed by Gu J et al. stratifies patients for chemoresistance prior to treatment initiation by utilizing grayscale US, elastography, and clinical data (AUC = 0.911; 95%CI: 0.814–0.979)^64^. This model can accurately predict NAC resistance and pCR before the commencement of therapy. The combined application of multiple modalities enhances predictive performance, optimizes patient stratification management, and averts ineffective treatment or excessive surgery, thus possessing substantial clinical translational value. Although existing research has validated the utility of AI models in circumventing overtreatment (such as ALN dissection) and futile chemotherapy, morphological alterations induced by NAC, such as lymph node fibrosis, continue to pose challenges for the identification of residual disease. Overall, AI-assisted efficacy evaluation systems are reshaping the management paradigm of NAC, and their clinical translation will be contingent upon larger-scale prospective validation.

### Challenges and limitations of ultrasound technology

The aforementioned studies confirm that US examination techniques can effectively predict pCR during the early stages of NAC and identify patient populations that are either sensitive or resistant to chemotherapy, thereby providing clinicians with a basis for the timely adjustment of therapeutic strategies. However, Although US technology has evolved from morphological assessment to encompass elastography and microstructural exploration, relevant research still generally confronts several common bottlenecks. First, post-NAC fibrotic scar tissue is difficult to distinguish from residual tumor foci, rendering precise differentiation between residual disease and treatment-related changes challenging and consequently impeding accurate evaluation of actual tumor dimensions. Second, the heterogeneity among molecular subtypes precludes the universal application of a single quantitative threshold across different subtypes; there is a lack of standardized criteria for defining the optimal peritumoral distance, establishing cut-off values for early functional parameters such as those derived from elastography, and determining the optimal assessment time window. Furthermore, although advanced US technologies, pathology-US cross-modal models, and DL models demonstrate considerable promise for future application, existing studies are predominantly limited to small sample sizes and single-center settings, the feature weights within the model decision-making process remain unclear—a manifestation of the “black box” problem—and certain technologies remain confined to validation in animal experiments. Concurrently, US is inherently operator-dependent, and inter-operator variability in image acquisition and interpretation may compromise the reproducibility and comparability of research findings. The aforementioned collective limitations dictate that prior to large-scale clinical translation, US techniques still require validation through prospective, multicenter studies, standardized operational protocols, and the support of interpretable AI technology.

## X-ray

X-ray techniques, particularly MG, by virtue of their standardized acquisition protocols, rapid imaging capabilities, and favorable cost-effectiveness, represent one of the primary modalities currently employed in clinical practice for the surveillance of NAC efficacy in BC patients^65^. This technique enables clear delineation of the tissue contrast between mammary glandular parenchyma and adipose tissue. It is utilized not only for baseline staging but, more critically, for the precise monitoring of the dynamic evolution of key morphological characteristics—including tumor size, morphology, density, and calcifications—through comparison of pre- and post-treatment images. Among these assessments, the evaluation of microcalcification clusters is particularly crucial^66^, as their presence may signify residual DCIS components, thereby providing important clues for the determination of pCR.

### Conventional X-ray techniques

DCIS is a non-invasive form of BC frequently detected during MG screening, where it typically manifests as clusters of microcalcifications^67^. A meta-analysis incorporating 31 articles indicated that following NCT, residual calcifications identified on MG may not exhibit a definitive correlation with the actual residual status of DCIS (29.2–60% of calcifications correspond to residual DCIS, whereas 38.5–62.2% of calcifications are pathologically confirmed as benign alterations)^9^. This finding suggests that reliance solely on microcalcification characteristics is insufficient for the effective assessment of NAC efficacy.

Although Mammographic Density (MD) on MG is an established risk factor for BC, its relationship with therapeutic response remains incompletely elucidated. Certain studies postulate that microenvironmental features associated with high mammographic density, such as abundant collagen deposition and elevated extracellular matrix pressure, may impede the effective delivery of chemotherapeutic agents, consequently resulting in diminished pCR rates, a phenomenon particularly pronounced among premenopausal patients^68,69^. However, an analysis of 292 BC patients undergoing NAC by Cullinane et al. concluded that baseline mammographic density (as per BI-RADS classification) does not serve as an independent predictor of pCR^70^. The findings of Rella R et al. further corroborate this conclusion; nonetheless, their investigation concurrently revealed a significant correlation between MD and prolonged DFS. The investigators hypothesize that this association may stem from the abundant collagenous stroma, elevated concentrations of growth factors, and enhanced immune cell infiltration characteristic of dense breast tissue, which collectively augment tumor chemosensitivity and thereby mitigate the risk of distant recurrence^14^. Ahn JH et al. employed an automated volumetric measurement approach to evaluate changes in breast density (ΔVbd%) among 357 patients. Through dynamic monitoring of density fluctuations, they observed that a reduction in breast density following NCT (ΔVbd% < –20%) was independently associated with lower pCR rates, particularly among patients with Luminal B-like and TNBC subtypes^71^. This density decline may be attributable to chemotherapy-induced ovarian suppression or post-treatment tissue fibrosis. The study implies that the emergence of decreased breast density during the treatment course may portend a suboptimal therapeutic response, thereby facilitating the early identification of patient cohorts that are less sensitive to chemotherapy^72^.

In summary, the potential utility of MD and its dynamic alterations as predictive biomarkers warrant further elucidation through additional investigations, and their underlying mechanisms of action as well as specific clinical applicability necessitate more in-depth exploration.

### Emerging X-ray techniques

Beyond conventional MG, emerging X-ray-based techniques are demonstrating considerable value in the assessment of therapeutic response. Digital breast tomosynthesis (DBT), through three-dimensional imaging that diminishes tissue superimposition, exhibits excellent performance in evaluating residual tumor dimensions, achieving an AUC of up to 0.84 for predicting pCR, particularly within the Luminal B subtype^73^. Contrast-enhanced mammography (CEM), an emerging modality that enhances visualization of tumor neovascularity through the intravenous administration of iodinated contrast media^74^, has shown prominent utility in the evaluation of NAC efficacy. Multiple investigations have established that the sensitivity and specificity of CEM surpass those of conventional MG, an advantage that is particularly pronounced among women with dense breast tissue^75–78^. In a prospective study encompassing 51 patients with invasive ductal carcinoma, Bernardi D et al. reported that CEM achieved a sensitivity of 81% and a specificity of 83% for the post-NAC assessment of pCR. Moreover, post-treatment tumor dimensions measured via CEM were slightly overestimated relative to pathological findings and demonstrated comparability to MRI. Concurrently, the study noted that delayed-phase CEM enhanced the detection rate of residual DCIS^76^. The work of Hogan MP et al. further substantiated the equivalence of CEM and MRI in the detection of residual disease, positing that CEM may serve as a viable alternative to breast MRI^77^. Collectively, these studies concur that CEM enhances both the sensitivity and specificity for detecting BC lesions relative to conventional MG, with this superiority being more pronounced in women with elevated breast density. Furthermore, CEM offers advantages including rapid acquisition, lower cost, and reduced examination time, thereby rendering it more amenable to large-scale clinical implementation and dissemination.

### Radiomics and deep learning techniques

In the domain of X-ray-based image analysis, DL models have demonstrated distinct advantages in their application^79^. Prior investigations have utilized multi-timepoint longitudinal mammographic images in conjunction with risk factors to construct DL models capable of predicting short- to long-term risk of BC development^80^. Förnvik D et al. prospectively enrolled 149 BC patients undergoing NAC and developed a DL model based on DBT. By analyzing sequential imaging data acquired at multiple timepoints relative to NAC (pre-NAC, after the second cycle, and after the sixth cycle), this model achieved precise prediction of pCR (AUC = 0.83), and its performance significantly surpassed that of conventional human assessment (AUC = 0.74, as evaluated by seven senior breast radiologists)^81^. Nevertheless, the test set in that study comprised only 30 cases, necessitating future validation through multicenter investigations with larger sample sizes. Yoon JH et al. employed DBT and AI-CAD as adjunctive tools to MG and, upon analyzing data from 314 patients who had undergone breast-conserving surgery, discovered that AI-CAD-assisted MG interpretation markedly reduced the recall rate and enhanced specificity (98.3%), albeit with low sensitivity (22.2%). Concurrently, the study revealed that DBT exhibited superior performance in detecting calcifications, whereas AI-CAD excelled in identifying masses or asymmetries, indicating that these two modalities possess complementary functionalities^82^. This synergy may alleviate challenges associated with postoperative breast imaging interpretation and bolster clinical diagnostic confidence. The research group led by Cai L retrospectively collated data spanning a decade, encompassing 720 BC patients who had received neoadjuvant systemic therapy. They innovatively developed a pretreatment multimodal ML algorithm integrating US, DBT, and clinical variables. This algorithm constructed a predictive model by extracting quantitative radiomics features from the tumor region and a 3-mm peritumoral zone, achieving an AUC of 0.81 (95% CI: 0.75–0.87)^83^. The study further noted that within the DBT modality, the original surface-to-volume ratio of the tumor was the most predictive feature parameter, suggesting that tumors exhibiting a spherical morphology are more likely to attain pCR. This predictive model holds promise for clinical application in the early identification of patient cohorts who may derive benefit from NT, as well as in providing decision-making support for the formulation of surgical de-escalation strategies (e.g., omission of surgery).

### Challenges and limitations of X-ray technology

X-ray examination, owing to its convenience, cost-effectiveness, and reliability, constitutes one of the imaging modalities employed for post-NAC surveillance in BC^84^; however, its clinical application is encumbered by several limitations. A primary concern pertains to the cumulative risk associated with ionizing radiation, which may potentially elevate the incidence of secondary malignancies, particularly among younger patients requiring repeated follow-up examinations or those with genetic susceptibility. Secondly, MG exhibits limited detection sensitivity within dense breast parenchyma, as a high-density background can attenuate the capacity to discern residual lesions or architectural distortion. While CEM enhances the conspicuity of tumor vascularity, it is constrained by a restricted field of view, lacks inherent three-dimensional imaging capability, and is contraindicated in individuals with hypersensitivity to iodinated contrast media. In the realm of radiomics and DL, although multimodal fusion models demonstrate promising predictive potential, their performance remains contingent upon the quality and heterogeneity of training data. Furthermore, these models have not yet undergone rigorous validation through large-scale, multicenter prospective studies, rendering their generalizability uncertain. Future endeavors should be directed toward the optimization of low-dose imaging techniques, the advancement of standardization and clinical translation for multimodal fusion models, and the further elucidation of the biological implications of mammographic density, thereby enhancing both the safety and accuracy of X-ray-based approaches in therapeutic response assessment.

## Magnetic resonance imaging (MRI)

MRI is the standard modality for assessing BC response to NAC. By virtue of its superior soft-tissue contrast and spatial resolution, MRI plays a pivotal role in the evaluation of tumor response following NT, as it is capable of concurrently providing both morphological and functional information pertaining to the tumor, thereby enabling precise assessment of tumor dimensions, extent of invasion, and angiogenic status^85^. The key characteristics and application scenarios of various MRI techniques are summarized in Table 3.

**Table 3:** Comparison of MRI techniques for assessing response to neoadjuvant therapy

Technical category	Technique name	Principle	Primary evaluation metrics	Advantages	Limitations	Clinical application scenarios	Refs
Morphological assessment	MRI	Utilizes physical properties of hydrogen nuclei in a strong magnetic field, excited by RF pulses, with signals reconstructed into images	T1, T2, etc.	High soft-tissue contrast and spatial resolution	High cost; Limited availability; Long scan time; Metal implant restrictions	Imaging “gold standard” for evaluating NT efficacy; Assessing ALN volume changes	^88,151^
Functional assessment	DCE-MRI	Assesses tissue blood flow perfusion using Gadolinium-based contrast agents	TIC, etc.	Very high soft-tissue resolution; Gold standard for tumor extent, multifocal lesions	Long scan time, high cost; Risk of Gadolinium deposition; Lower specificity for non-mass enhancement	Accurate preoperative staging; Assessing feasibility of breast-conserving surgery; Detecting ALN metastasis	^90,93^
	DWI	Detects restricted Brownian motion of water molecules, reflecting cellular density	ADC value	No contrast agent needed; Functional imaging for early monitoring of necrosis/apoptosis	ADC value affected by scan parameters; Intratumoral heterogeneity; Non-linear relationship with pathological response	Early prediction of treatment response (after 1–2 cycles); Monitoring cytotoxic therapy effects	^94,99^
	MRE	Quantifies tissue mechanical properties to reflect elasticity	TSR, PAR	Non-invasive; Multi-parameter assessment; Good reproducibility	Limited spatial resolution; Preliminary exploration stage	Early prediction of NT effectiveness during treatment	^17^
Metabolic and molecular imaging assessment	CEST(APT)	Saturates protons at specific frequencies, monitors chemical exchange with free water, generating magnetization transfer contrast	LAC/PYR ratio, etc.	Non-invasive detection of specific metabolites (proteins, glycogen) concentration and pH; Provides molecular-level information	Weak signal, high demand for magnetic field homogeneity; Long scan time; Complex quantitative analysis	Research stage; Monitoring tumor protein metabolism, acidosis, and other treatment-related biochemical changes	^102,105^
	Hyperpolarized ¹³C-MRI	Hyperpolarizes ¹³C-labeled substrates (e.g., pyruvate) to greatly enhance MR signal, tracks metabolic conversion in real-time after IV injection	LAC/PYR ratio, etc.	Non-invasive, real-time, dynamic visualization of key metabolic pathways in vivo; Ultra-high sensitivity	Technically complex/expensive; Short hyperpolarization state lifetime; Requires dedicated hardware	Cutting-edge research tool for very early (within hours) monitoring of metabolic reprogramming, predicting treatment response	^23^

*TIC* time-intensity curve, *TSR* tumor stiffness ratio, *PAR* phase angle ratio, *LAC/PYR* lactate-to-pyruvate ratio.

### MRI Technology and DCE-MRI

Reig B et al. emphasized in their review that MRI currently represents the most accurate imaging modality for evaluating NAC response^86^. The prospective, multicenter ACRIN 6657 trial conducted by Scheel JR et al. confirmed that the longest diameter of the mass measured on MRI is the most precise method for assessing residual disease and predicting pCR after NAC and prior to surgery. This holds particularly true for patients with multifocal masses, and the accuracy of MRI assessment was not significantly influenced by molecular subtype or mammographic density^87^. Cattell RF et al. were the first to systematically evaluate the predictive value of ALN volume changes during the course of NAC, with data acquisition performed at four distinct timepoints. Their investigation revealed that baseline ALN volume does not predict pCR; however, during the early treatment phase, the pCR group exhibited a more rapid and pronounced reduction in volume, demonstrating high discriminatory capacity. The change in ALN volume may thus serve as an early predictor of NAC efficacy. Notably, for patients with large baseline lymph nodes (volume ≥3 mL), the combination of ALN volume change with breast tumor volume changes significantly enhanced predictive performance (AUC = 0.87). Patients with HR-negative, TNBC, and HER2+ subtypes presented with relatively larger ALN volumes, suggesting that these subtypes are more prone to lymph node metastasis but may also derive greater benefit from NAC^88^. This study substantiates that accurate MRI assessment of ALN can effectively reduce the need for unnecessary sentinel lymph node biopsy.

DCE-MRI enables quantitative evaluation of tissue perfusion, vascular permeability, and microcirculatory characteristics through intravenous administration of contrast material (e.g., gadolinium-based agents) and continuous monitoring of its kinetic distribution. Kim SY et al. acquired DCE-MRI images from 487 BC patients after NAC and prior to surgery across three phases: early, conventional delayed, and late delayed phases. Their analysis demonstrated that the early phase is more suitable for predicting pCR but may underestimate the true total tumor dimensions. In contrast, delayed-phase MRI exhibited the highest concordance with pathological tumor size and was identified as the optimal temporal phase for assessing residual tumor extent. Consequently, clinical evaluation should integrate imaging information from both temporal phases to ensure accurate judgment^89^. This study also observed significant underestimation of tumor size on MRI for lobular carcinoma and the ER+/HER2– subtype, a finding consistent with the sonographic conclusions reported by ref. ^43^, thereby indicating that clinical practice for patients with this subtype may warrant consideration of expanded surgical resection margins based on imaging findings. In addition to monitoring alterations in tumor size, the background breast parenchyma also merits attention. The investigation by Arefan D et al. sought to determine whether quantitative measurement of BPE could serve as a non-invasive imaging biomarker for predicting recurrence risk in patients with ER+/HER2–, node-negative BC. DCE-MRI examinations were performed preoperatively, and the analysis revealed that quantitative BPE was significantly associated with a high risk of recurrence in ER+ disease (ipsilateral side, *P* < 0.001–0.02), with a stronger association observed in premenopausal patients. The integration of BPE parameters with tumor radiomics features enabled the high-accuracy differentiation of patients at high risk from those at low/intermediate risk (AUC = 0.94; 95% CI: 0.90–0.98)^90^. This model provides a non-invasive and cost-effective alternative for recurrence risk assessment in resource-limited settings and holds promise as a substitute for more expensive standard recurrence risk scoring tools such as Oncotype DX.

Beyond conventional DCE-MRI, a growing body of research is focusing on fast DCE-MRI. This technique possesses higher temporal resolution, which may allow for more accurate capture of early tumor perfusion characteristics, and it does not necessitate the use of dedicated post-processing software^91^. The study by Cao Y et al. found that ultrafast DCE-MRI parameters can effectively predict pCR early in the course of NAC (after the second cycle), and that changes in these parameters over time exhibit stronger predictive power than single-timepoint measurements, underscoring the superiority of a dynamic monitoring strategy over static assessment^92^. Musall BC et al. utilized FTV measured by fast DCE-MRI and demonstrated that it could accurately predict pCR in TNBC patients after NT, achieving an AUC of 0.85 (95% CI: 0.75–0.96) by the fourth treatment cycle^91^. In the prospective study by Guirguis MS et al., which enrolled 287 patients with stage I–III TNBC, DCE-MRI images were acquired at baseline, after the second cycle, and after the fourth cycle, with manual delineation of three-dimensional tumor volume. The results indicated that the percentage reduction in tumor volume measured manually on DCE-MRI could predict pCR with high accuracy as early as after the second cycle (AUC ≈ 0.82), exhibiting predictive capability comparable to that of FTV^93^. This methodology provides a basis for early therapeutic regimen adjustments in TNBC patients and, owing to its independence from specialized software, is more readily amenable to widespread adoption and application in clinical practice.

### DWI-related techniques and MRE technology

DWI is an MRI technique that does not require contrast agent administration. Its signal intensity is inversely proportional to the diffusivity of water molecules, and the ADC quantitatively reflects the degree of restricted water diffusion, thereby enabling non-invasive assessment of treatment-induced alterations in the tumor microenvironment. A growing body of evidence substantiates that, in the evaluation of NAC efficacy, DWI is capable of providing early monitoring of pathological changes induced by therapy, including disruption of tumor cell membrane integrity, reduction in cellular density, and necrosis^20,94,95^. Although Surov A et al. reported that baseline ADC values do not predict NAC response in BC patients^96^, a prospective study by Pereira NP et al. confirmed that dynamic changes in ADC (ΔADC%) during early treatment reflect pCR earlier than morphological indicators^95^. In a multicenter trial, Partridge SC et al. demonstrated that ΔADC at week 12 of NAC is an independent predictor of pCR, achieving an AUC of 0.76 (95% CI: 0.62–0.89; *P* < 0.001), particularly within the HR+/HER2– subtype. Concurrently, this study also found that changes in FTV measured by DCE-MRI exhibited predictive performance for pCR similar to that of ΔADC% (AUC = 0.63–0.68)^20^. Zhao R et al. further integrated DCE-MRI and DWI to construct a predictive model (after the second cycle), which also demonstrated excellent performance (AUC = 0.939–0.944)^97^, thereby facilitating early treatment regimen adjustment. The aforementioned studies indicate that the predictive utility of ADC for pCR is contingent upon its dynamic post-treatment evolution. The underlying mechanism may involve NAC-induced disruption of cell membrane integrity and reduction in cellular density^98^, leading to an increase in ADC values; however, as this process requires time, dynamic monitoring holds greater significance than a single baseline measurement.

To further overcome the limitations inherent to conventional DWI, such as parameter overlap and inadequate reflection of tumor microstructural heterogeneity, time-dependent diffusion MRI has been developed, enabling the quantification of microstructural parameters, including cell diameter and density. A prospective investigation by Wang X et al. revealed that time-dependent diffusion MRI can non-invasively predict molecular subtypes prior to the initiation of NAC, with particularly notable performance in the HER2-enriched subtype (AUC = 0.85; 95% CI: 0.80–0.89; *P* < 0.001). Moreover, cellular density was identified as the optimal single predictor of pCR (AUC = 0.84; 95% CI: 0.77–0.90), and the integration of PR and HER2 status further enhanced model performance (AUC = 0.88; 95% CI: 0.62–0.89; *P* < 0.001)^99^. This technique represents the first achievement of non-invasive quantification of cellular density and elucidated that patients who attain pCR typically exhibit baseline characteristics of lower cellular density and larger cell diameter, thereby offering a novel perspective for therapeutic response prediction.

IVIM-DWI and DKI, as advanced functional MRI techniques predicated on non-Gaussian diffusion models, substantially enhance the capacity to quantify microstructural heterogeneity within biological tissues by overcoming the limitations inherent to the conventional mono-exponential DWI model. Chen X et al. integrated parameters derived from DCE-MRI, DKI, and IVIM to predict NAC efficacy in patients with IDC prior to treatment initiation, achieving an AUC of 0.969 (95% CI: 0.901–0.995), a sensitivity of 94.12%, and a specificity of 90.70%. Moreover, the incorporation of imaging parameters with clinical features was found to further augment the accuracy of predicting NAC response^100^. Nevertheless, this study was constrained by a relatively small sample size of 77 patients and its restriction to IDC; future investigations are warranted to extend these findings to other molecular subtypes. In a prospective study, Gong X et al. compared the predictive capacity for pCR among multi-b-value diffusion MRI models (including DWI, IVIM, and DKI) at various timepoints during the course of NAC. They observed that the DKI model accurately predicted pCR as early as the initial phase of NAC (after the second cycle), attaining an AUC of 0.90 (95% CI: 0.84–0.94). The authors posited that DKI may be more suitable than IVIM for reflecting the complex non-Gaussian diffusion microenvironment characteristic of BC, thereby suggesting that DKI holds promise as a preferred imaging modality for the early assessment of BC NAC response^101^. Advanced diffusion models predicated on histogram analysis, through multiparametric and multidimensional quantitative evaluation, furnish more precise imaging biomarkers for monitoring alterations within the tumor microenvironment.

MRE provides a unique biomechanical perspective for assessing tumor therapeutic response by quantifying the mechanical properties of tissue. This emerging technique enables non-invasive measurement of stiffness alterations within tumor tissue, thereby offering crucial supplementary information for clinical decision-making. Recent findings reported by Sinha AP et al. demonstrated a statistically significant difference in post-treatment TSR between complete and partial responder cohorts (*P* < 0.001), indicating that alterations in tumor mechanical characteristics are intimately associated with treatment response. More notably, phase angle ratio (PAR), a key index reflecting tissue viscoelasticity, exhibited exceptional predictive performance as early as the initial treatment phase (after the second cycle). These data not only substantiate the correlation between MRE-derived parameters and pCR but further corroborate that when MRE is integrated with DCE-MRI, the specificity for identifying complete responders can be elevated to 96%, while concurrently maintaining a high sensitivity of 94%^17^. It is imperative to acknowledge, however, that the current application of MRE in the domain of tumor therapeutic response assessment remains at a preliminary exploratory stage. Its predictive value across diverse tumor types awaits validation through large-scale clinical investigations, which will constitute a pivotal direction for future research in oncological biomechanical imaging.

### CEST imaging and hyperpolarized ¹³C-MRI

The transmembrane cycling of water molecules constitutes a critical biological process in cellular metabolism, and its aberrant enhancement is intimately associated with tumor invasiveness and metabolic activity. However, conventional MRI techniques are inherently limited in their capacity to directly evaluate such microscopic dynamic alterations. The advent of CEST imaging has provided a groundbreaking solution to this challenge. By detecting the signal exchange between intracellular exchangeable protons (e.g., amide protons) and bulk water protons, this technique enables non-invasive assessment of tumoral water molecule transmembrane cycling and metabolic activity^102^. Among the CEST variants, APT imaging is currently the most extensively investigated, demonstrating sensitivity to intratumoral protein content and microenvironmental pH variations^103^. This distinctive molecular imaging mechanism has positioned APT as a prominent focus of research in the evaluation of tumor therapeutic response.

In the context of BC research, Krikken E et al. employed 7 T APT CEST MRI to assess early response to NAC in nine BC patients, demonstrating that this technique could detect metabolic alterations associated with pathological response at a very early stage of treatment (3 weeks). The directional changes in signal intensity exhibited a degree of predictive value^104^. Zhang S et al. further reported that a decline in APT-weighted signal intensity in TNBC patients after the second cycle of NT may be indicative of pCR; however, the limited specificity of this index suggests that APT as a standalone modality is insufficient for achieving precise individual discrimination^105^. Collectively, these studies support the utility of APT imaging in providing non-invasive early monitoring of therapeutic efficacy and guiding strategic adjustments.

Although CEST techniques, via endogenous contrast mechanisms such as APT imaging, enable non-invasive assessment of the molecular environment, their imaging specificity and sensitivity remain constrained by the intrinsic properties of the exchangeable proton pool^106^. To surmount this technical bottleneck, targeted imaging strategies predicated on exogenous molecular probes have been developed. For the early monitoring of tumor metabolic alterations, hyperpolarized ¹³C-MRI exhibits unique advantages. Research by Woitek R et al. demonstrated that by measuring the ratio of pyruvate to lactate (LAC/PYR), this technique can predict pCR in BC patients within 7–11 days following NAC initiation, wherein an elevated ratio portends a favorable therapeutic response. Notably, this metabolic ratio is closely correlated with the expression levels of the pyruvate transporter MCT1 and lactate dehydrogenase A, thereby elucidating, at a molecular level, the dynamic shifts in tumor glycolytic activity and hypoxic status^23^. This transformative advancement has collectively propelled a paradigm shift in molecular imaging from macroscopic morphological assessment to microscopic metabolic surveillance. Nonetheless, its clinical translation is confronted with several challenges. Firstly, the biocompatibility, pharmacokinetics, and long-term safety profile of exogenous probes warrant further rigorous validation to facilitate clinical adoption. Secondly, the technical complexity and substantial cost associated with hyperpolarized ¹³C-MRI impede its widespread dissemination, underscoring the need for future optimization of imaging protocols and the development of more stable hyperpolarization techniques. As precision medicine continues to evolve, these advanced molecular imaging modalities hold considerable promise as integral tools for individualized therapeutic decision-making, with the ultimate objective of refining the clinical management of oncology patients.

### Radiomics and deep learning technology

**Fig. 3:**
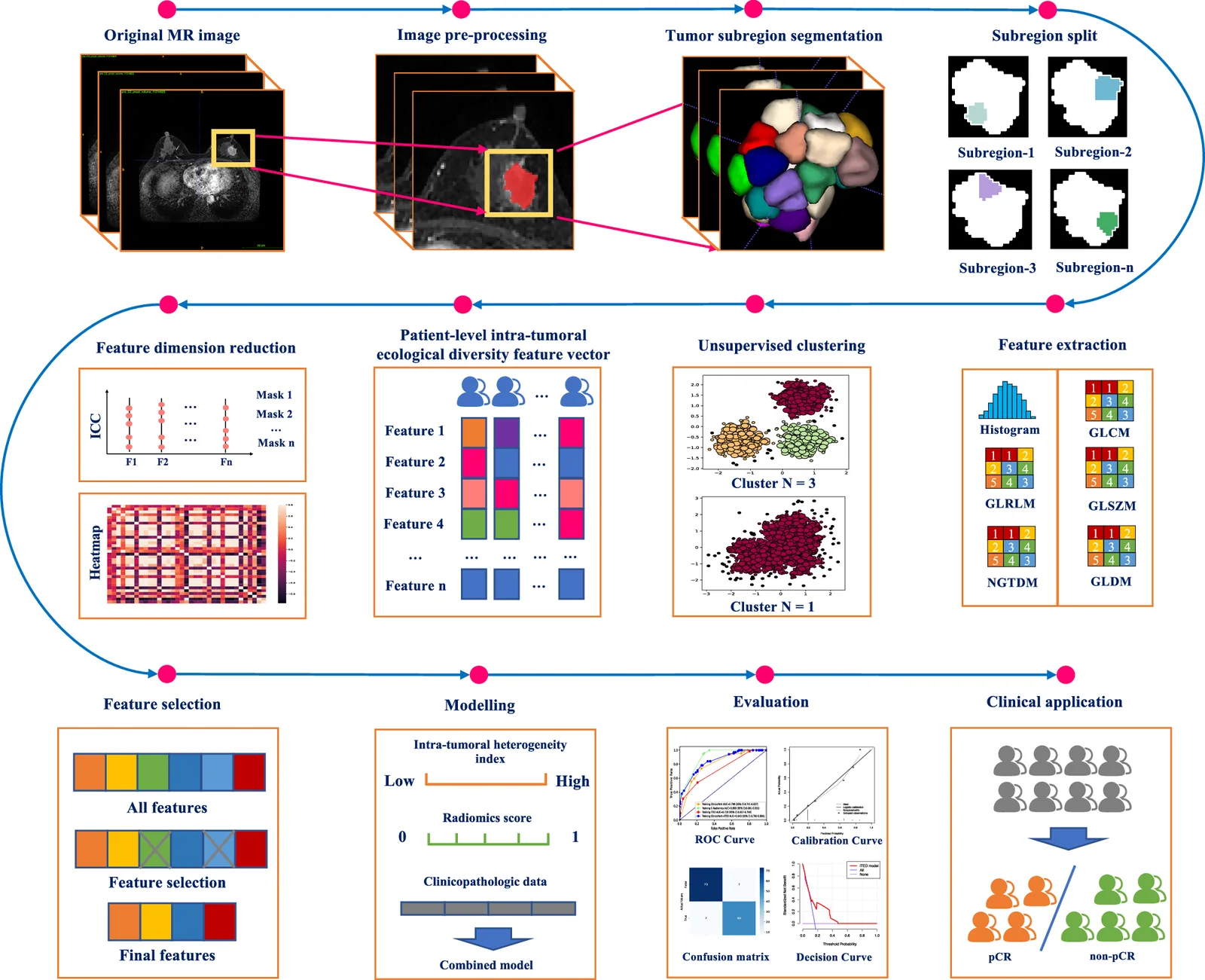
Schematic shows the workflow of the proposed breast cancer intratumoral heterogeneity (ITH) assessment method used in this study. First, the intratumoral subregions were segmented from manually delineated tumor regions. Second, an intratumoral ecological diversity feature vector was generated per patient. Third, feature selection processes were performed to select the features with predictive power. Finally, a combined model constructed using the ITH index, conventional radiomics score, and clinicopathologic data was evaluated using multiple external test datasets. GLCM gray-level co-occurrence matrix, GLDM gray-level dependence matrix, GLRLM gray-level run-length matrix, GLSZM gray-level size-zone matrix, ICC intraclass correlation coefficient, NGTDM neighboring gray-tone difference matrix, pCR pathologic complete response, ROC receiver operating characteristic. Copyright 2023, Radiology, CC BY 4.0.

MRI-based radiomics and AI models have emerged as powerful tools for predicting pCR. As presented in Table 4, representative studies illustrate an evolutionary trajectory from conventional ML to DL and further towards multi-omics integration. Early investigations constructed predictive models by extracting a multitude of morphological and textural features, often in conjunction with algorithms such as logistic regression. For instance, a retrospective study by Shi Z et al. achieved non-invasive quantification of ITH in BC via MRI and validated its capacity to predict pCR prior to treatment initiation (a schematic flowchart of the model is depicted in Fig. 3). Their predictive model, which integrated clinicopathological variables with conventional radiomics, demonstrated excellent performance across four independent datasets (AUC = 0.83–0.90), exhibiting robust generalizability. The ITH index, which reflects the ecological diversity of the tumor microenvironment, was identified as a potent predictor independent of molecular subtyping^107^. This investigation was the first to introduce the concept of “intratumoral ecological diversity” into the MRI domain for predicting NAC response. By quantifying intratumoral heterogeneity through clustering analysis of tumor subregions, the study achieved a substantial enhancement in predictive performance. This methodology possesses the potential for transferability to other imaging modalities and exhibits broad applicability. Zhang J et al. amassed data from 6991 BC patients across four centers, totaling 14,472 images (including MG, US, and MRI). Prior to the initiation of therapy, a HER2 MAP model was first constructed to predict HER2 status, followed by the development of a Response MAP model to forecast pCR. In both the test set and three external validation cohorts, the pCR prediction AUC of this model significantly surpassed that derived from core needle biopsy results^108^. This model substantiates that the HER2 MAP model can non-invasively and accurately predict HER2 status, outperforming biopsy, particularly in capturing intratumoral heterogeneity. Nevertheless, this model was exclusively based on a single modality, DCE-MRI, and the study population was predominantly of Asian ethnicity, introducing potential selection bias; consequently, validation across more diverse populations is warranted in future research. The team led by Bitencourt AGV integrated pretreatment MRI clinical features, DCE-MRI radiomics features, and ML algorithms to achieve high-precision discrimination between protein overexpression (IHC 3+) and gene amplification (FISH positive) in HER2-overexpressing BC, attaining an accuracy of 97.4%. Concurrently, the study observed that radiomics heterogeneity parameters were elevated within the pCR cohort, suggesting an association between greater radiomics heterogeneity and a more favorable therapeutic response. This observation contrasts with the clinical implications of pathological heterogeneity, implying that the heterogeneity reflected by radiomics and pathology may correspond to distinct dimensions of biological characteristics^109^.

**Table 4:** Summary of key predictive models for pCR in breast cancer NAC

Model	Cohort	Clinical pathology + ITH + C-radiomics^107^	pCR prediction Model^109^	Tumor + peri VOI radiomic model^111^	rCR model^110^	MLRL model^112^	Synthetic MRI (SyMRI)^113^	Deep Learning Signature (DLS)^116^	Clin-SHR^115^
Study design		Retrospective	Retrospective	Retrospective	Prospective	Retrospective	Prospective	Retrospective	Retrospective
Timepoint of action		Before treatment	Before treatment	Before treatment	After treatment	pre-NAT or mid-NAT or post-NAT (random pairing of two timepoints)	Before treatment and after four cycles	Before treatment	Before treatment and After two cycles
Modeling approaches		Multivariable logistic regression	Coarse decision trees	Supporting vector machine (SVM)	Multivariable logistic regression	MLRL	Logistic regression model	3D CNN	XGBoost
Feature Count		ITH features: 5; C-radiomics features: 105	6	Top 30 features each from T1 + C, T2WI, DWI-ADC	32	-	930	512	pre-NAT SHR (7) + mid-NAT SHR (8) + clinical characteristics (3)
Number of cases (Centers)		1589(4)	311(1)	448(2)	259(3)	1992(21)	181(1)	139(1)	2279(12)
External validation		Yes	No	Yes	Yes	Yes	No	No	Yes
AUC (95% CI)	Train	0.90 (0.85, 0.95)	-	0.980	0.810 (0.74, 0.87)	0.843 (0.806, 0.876)	0.78	-	0.928 (0.903, 0.953)
	Val	0.83–0.87	-	0.920	0.830 (0.75, 0.92)	0.816 (0.773, 0.857)	0.72 (0.58, 0.87)	0.958	0.863
Accuracy	Train	0.84	0.851	0.990	-	-	-	-	-
	Val	0.79–0.81	0.839	0.830	-	-	-	0.923	0.811
Sensitivity	Train	0.79	0.874	0.960	0.780	0.819	-	-	0.874
	Val	0.65–0.72	0.865	1.000	0.830	0.746	-	0.933	0.771
Specificity	Train	0.86	0.816	0.990	0.840	0.769	-	-	0.871
	Val	0.84–0.85	0.800	0.800	0.840	0.746	-	-	0.834
Key limitations		Manual segmentation; utilization of a single phase only; biological mechanisms not explored	Single-center; heterogeneity of external institutional images; small sample size in the FISH cohort; restricted to HER2+	Selection bias; manual segmentation; single ethnicity; relatively low pCR rate	Limited sample size; heterogeneity of parameters; DCIS not included; absence of diffusion/ultrafast imaging	Reliance on a single DCE-MRI sequence; lack of text standardization; high computational complexity	Single-center; fat signal not suppressed; manual segmentation; single scanner; restricted to TNBC	Single-center; small sample size; lack of external validation; imbalance among subtypes; manual segmentation	Predefined segmentation algorithm; absence of prospective validation; reliance on a single DCE-MRI sequence; heterogeneity of the cohort

Train training cohort, Val validation cohort.

Beyond pretreatment investigations, a prospective multicenter study by Ramtohul T et al. conducted post-treatment DCE-MRI examinations in TNBC patients and found that rCR on MRI demonstrated robust predictive value for pCR (AUC = 0.83; 95% CI: 0.75–0.92, PPV = 90%). Notably, within the subgroup characterized by rCR, negative lymph node status, and Ki67 > 30%, the false-positive rate was a mere 3.5%, indicating that these patients may represent ideal candidates for future “surgery omission” clinical trials. Further analysis revealed that for patients exhibiting residual enhancement, simple shape descriptors and first-order radiomics features could assist in differentiating inflammatory changes from genuine residual tumor^110^.

Technological evolution is further manifested in the exploratory analysis of the peritumoral region and the deepening application of DL. Li C et al. demonstrated that a model integrating features from both the tumor and a 10-mm peritumoral region (AUC = 0.92) prior to treatment outperformed models relying solely on intratumoral features^111^. However, the 10-mm peritumoral margin adopted in this study diverges from those utilized in related US and X-ray investigations. This discrepancy indicates that while the peritumoral microenvironment possesses unequivocal predictive value, the optimal assessment boundary remains unstandardized across different imaging modalities, and its clinical applicability and reliability warrant further in-depth exploration. In the field of DL, Gao Y et al. proposed a time-based model termed multimodal longitudinal representation learning (MLRL) and constructed the largest multimodal dataset to date for monitoring BC NT response. The internal dataset comprised 3719 DCE-MRI scans from 1420 patients, while the external validation cohort was derived from the I-SPY 2 dataset, encompassing 2516 examinations acquired at three distinct timepoints: pretreatment, mid-treatment, and post-treatment. The results demonstrated that the MLRL model achieved AUC values of 0.843 (95% CI: 0.806–0.876) and 0.816 (95% CI: 0.773–0.857) on the internal and external datasets, respectively, significantly outperforming existing methodologies^112^. This model not only accurately predicts therapeutic response but also enables the visualization of tumor progression, thereby harboring the potential to advance the automation of clinical temporal decision-making and serving as a promising tool for individualized BC treatment. Li Z et al. collected data from 1145 patients with ALN-positive BC across six Chinese medical centers over a five-year period and leveraged DL technology to construct FAIS-DL. The study revealed that this system could accurately predict axillary pCR prior to treatment (AUC > 0.93), with performance significantly exceeding that of single-region models and clinical models. Furthermore, the investigation found that a higher FAIS-DL score correlated with activation of the immune microenvironment, thereby providing a biological rationale for the model’s predictive output^19^. Clinical implementation of this model could potentially reduce the rate of unnecessary ALND from 47.9 to 6.8%, consequently leading to a substantial decrease in associated surgical complications. FAIS-DL represents the first multicenter, prospectively validated system for predicting axillary pCR and holds considerable promise for guiding de-escalation strategies in axillary surgery and mitigating postoperative morbidity.

It is noteworthy that SyMRI, by virtue of its distinct advantages, has furnished novel avenues for radiomics research. SyMRI can generate quantitative parameter maps without the administration of contrast agents. Its rapid acquisition characteristics effectively mitigate motion artifacts while preserving consistent voxel geometry, thereby creating optimal conditions for multiparametric integrated analysis. A study by Hwang K-P et al. confirmed that a predictive model constructed from SyMRI-derived quantitative T1 maps acquired during the mid-course of NAC (fourth cycle) in TNBC patients achieved an AUC of 0.72 (95% CI: 0.58–0.87)^113^. This technique obviates the need for contrast material and necessitates only a single acquisition, thereby circumventing potential risks associated with contrast agents and rendering it particularly suitable for young TNBC patients who require repeated follow-up monitoring.

The current research frontier is increasingly oriented toward longitudinal dynamic surveillance, multimodal imaging integration, and the fusion of multi-omics data. In a large-scale, multicenter, prospective radiogenomics investigation, Lin P et al. leveraged data from the I-SPY 2 trial, which included 383 BC patients with complete baseline gene expression profiles and multiparametric MRI data. Their analysis revealed significant correlations between the BC-associated genes NCS1, CD83, and ICAM3 and DCE-MRI parameters, which demonstrated moderate predictive utility, whereas the BFAR gene exhibited no significant predictive value. Furthermore, these imaging features were associated with tumor immune phenotypes, suggesting their potential applicability in predicting response to immunotherapy^114^. Radiogenomics approaches confer biological interpretability upon MRI parameters, thereby facilitating the formulation of individualized therapeutic strategies. The research group led by Huang YH, through a 12-center cohort study encompassing 2279 cases, innovatively integrated longitudinal dynamic MRI spatial habitat radiomics (SHR) features acquired at baseline and after the second cycle with transcriptomic and single-cell RNA sequencing data to construct a Clin-SHR multimodal model. This model demonstrated excellent performance in external validation, immunotherapy, and multi-omics cohorts (AUC = 0.863–0.888). Concurrently, the study identified a significant correlation between elevated Clin-SHR scores and B-cell infiltration, implying that B cells may play a crucial role in NAT efficacy^115^. This work represents the first elucidation of the immunobiological basis of radiomics features at the single-cell level, thereby not only providing a novel tool for non-invasive early response assessment but also establishing a methodological foundation for the clinical translation of combined imaging and immunotherapy strategies.

The aforementioned radiogenomics studies are predominantly predicated on transcriptomic data; however, proteins, as the direct executors of biological functions, reside closer to the terminal events of biological processes. In light of this, Duan J et al. pioneered the construction of a DL radiomics signature (DLS) model based on pretreatment multiparametric MRI, which achieved high-accuracy prediction of pCR following NAC (AUC = 0.958). Proteomic analysis further elucidated the biological pathways associated with pCR, encompassing key processes such as mitochondrial metabolism, vesicle trafficking, protein localization, and cytoskeleton-dependent transport. The DLS exhibited significant associations with these pathways, thereby furnishing a molecular-level biological rationale for the predictive capacity of the DL model^116^. A comparison of the pCR prediction performance by AI models in representative studies is presented in Table 4. Collectively, these investigations robustly demonstrate that ML-based radiomics models have become pivotal tools for predicting pCR, capable not only of optimizing therapeutic decision-making but also of holding considerable promise for enhancing the rate of successful BCS and ultimately facilitating the dynamic refinement of individualized treatment strategies.

### Challenges and limitations of MRI technology

MRI exhibits notable advantages in the multiparametric and multimodal assessment of NAC efficacy; nevertheless, its clinical application continues to encounter multifaceted challenges. Primarily, the high procedural cost, limited accessibility of equipment, and protracted scanning duration constrain its widespread utilization in primary healthcare settings and scenarios necessitating repeated follow-up examinations. Secondly, DCE-MRI relies on gadolinium-based contrast agents, which are predominantly excreted via the renal pathway. This dependence is associated with a potential risk of nephrogenic systemic fibrosis and raises concerns regarding cerebral gadolinium deposition. Although the safety profile of newer contrast agents has improved, their long-term biological sequelae warrant continued surveillance and evaluation. In the domain of functional imaging, ADC values derived from DWI are susceptible to interference from various factors, including magnetic field strength, acquisition parameters, and tumor heterogeneity. A lack of standardization across different institutions consequently undermines, to a certain extent, the clinical utility of quantitative comparisons. Advanced techniques such as IVIM, DKI, and MRE can provide more comprehensive microstructural information; however, their complex sequences, time-intensive post-processing requirements, and the absence of unified analytical standards have precluded their routine clinical implementation. Molecular imaging modalities, including CEST and hyperpolarized ¹³C-MRI, remain at an investigational stage and confront translational barriers pertaining to the biosafety of exogenous probes, technical stability, and substantial cost. Although radiomics and AI models have demonstrated exceptional predictive performance, they are encumbered by issues of insufficient interpretability, the “black box” nature of their decision-making processes, and a reliance of model generalizability on the quality and heterogeneity of training data. Moreover, the preponderance of current studies consists of single-center retrospective analyses, underscoring an urgent need for validation through multicenter, prospective clinical investigations. Additionally, MRI is associated with a certain degree of diagnostic risk, manifesting as false-positive and false-negative findings, in the detection of minimal residual disease, the interpretation of non-mass enhancement lesions, and the differentiation between post-treatment fibrosis and viable tumor tissue. Future research endeavors should be directed toward advancing the standardization of scanning protocols, optimizing rapid imaging techniques, exploring contrast-free functional imaging alternatives, conducting large-scale multicenter collaborative studies, and enhancing the interpretability and transparency of AI models. These efforts are essential for further augmenting the precision and clinical applicability of MRI in the evaluation of NT efficacy.

## Positron emission tomography/ computed tomography (PET/CT)

Since its advent in the late 20th century, PET/CT has emerged as an indispensable and pivotal tool in clinical oncology, owing to its distinct advantages in metabolic imaging. It is extensively employed across multiple critical domains, including tumor staging^117^, treatment planning^118^, therapeutic response assessment^119^, and prognostic surveillance^120^. PET/CT integrates two modalities—PET, which furnishes functional metabolic information, and CT, which provides anatomical localization—thereby enabling precise lesion localization and characterization through radionuclide tracing (e.g., ¹⁸F, ¹¹C) and image fusion.

### ^18^F-FDG PET/CT

This technique can reflect the metabolic status of tissue by detecting the uptake of ¹⁸F-fluorodeoxyglucose (FDG). The aberrant accumulation of FDG within tumor lesions is partly attributable to the upregulation of glucose transporter proteins in malignant cells^121^. This modality not only provides qualitative information regarding tumor metabolic activity but also, through quantitative parameters such as SUV, MTV, and TLG, enables an objective reflection of the tumor’s intrinsic biological behavior, thereby demonstrating substantial value in predicting NT efficacy.

Multiple studies have substantiated that semiquantitative parameters derived from PET/CT are intimately associated with pCR following NAC^122,123^. For instance, Alshaibani N et al. compared the accuracy of MRI and PET/CT in predicting therapeutic response. Through the analysis of images from 138 BC patients who underwent NAC prior to BCS, they ascertained that PET/CT exhibited superior sensitivity (93.8%) in predicting pCR, rendering it more suitable for identifying patients with a favorable response to NAC. Conversely, MRI demonstrated higher specificity (90.4%) in detecting residual disease, thus being more appropriate for delineating the extent of residual lesions in patients with an incomplete response^122^. The study by van Amstel et al. indicated that baseline PET/CT possesses limited predictive value for ALN status, yet molecular subtype remains a pivotal determinant in guiding individualized therapy^124^.

In the realm of radiomics exploration, the investigation by Groheux et al. was the first to systematically integrate pretreatment multimodal data (incorporating clinical, pathological, genomic, PET/CT, and radiomics data) for pCR prediction in TNBC, achieving an AUC of 0.82^125^. This research strategy circumvented the conventional reliance on interim PET; however, it was constrained by a limited sample size and the absence of external validation. Conversely, the study by Oliveira et al. revealed that first-order radiomics features extracted from baseline PET/CT could not effectively predict pCR across the majority of BC subtypes^126^. In summary, although PET/CT exhibits high sensitivity in the prediction of pCR, no other demonstrably superior advantages have been identified. The subsequent development of radiomics models has yielded a modest improvement in predictive capacity; nevertheless, the body of relevant literature remains circumscribed and is predominantly characterized by small sample sizes, thereby necessitating further investigation to ascertain the generalizability of these models.

### ⁶⁸Ga-FAPI PET/CT

Beyond conventional ¹⁸F-FDG PET/CT, the development of novel molecular probes in recent years has expanded the armamentarium for oncologic therapeutic response assessment. Among these, ⁶⁸Ga-labeled fibroblast activation protein inhibitor (⁶⁸Ga-FAPI) PET/CT has demonstrated distinct advantages in the detection of BC. Chen L et al. investigated the capacity of ⁶⁸Ga-FAPI PET/CT to predict pCR and tumor regression patterns at two timepoints: after the second cycle of NAC and preoperatively. Their findings indicated that this technique could accurately predict pCR at both junctures (AUC = 0.848–0.978). The study concurrently revealed that SUVmax (the highest SUV value within the lesion, representing the most metabolically active region), the absolute value of the TBR (the ratio of tumor SUVmean to that of the contralateral normal breast), and the magnitude of their decline were pivotal indicators for predicting pCR and concentric tumor shrinkage. Furthermore, this investigation noted that, in comparison to FDG PET/CT, ⁶⁸Ga-FAPI PET/CT exhibited greater sensitivity in detecting low-grade tumors, lobular carcinoma, and small lesions, and was less susceptible to fluctuations in blood glucose levels and inflammatory processes^127^. However, this study was constrained by a small sample size (only 22 cases).

Within the domain of molecular imaging, HER3 PET imaging offers a novel perspective for elucidating the intrinsic mechanisms governing BC therapeutic response. A prospective clinical study conducted by Sinevici N et al. explored the dynamic alterations in HER3 protein expression levels across different molecular subtypes of BC before and during NT. The investigation revealed pronounced heterogeneity in HER3 protein expression within BC, with the highest levels observed in the HER2+ subtype and the lowest in TNBC. Early changes in HER3 expression were noted following NT, and the upregulation of HER3 was correlated with the establishment of an immunosuppressive microenvironment, accompanied by enhanced cell proliferation signaling, thereby implicating its potential involvement in therapeutic resistance^128^. These observations not only substantiate the potential of HER3 as a prognostic biomarker—enabling early identification of resistance and guiding precise strategies for combined anti-HER3 targeted therapy—but also underscore the unique value of molecular imaging techniques in the real-time surveillance of tumor biological behavior. Nonetheless, at the present stage, these novel technologies remain in the exploratory research phase. As molecular imaging and precision medicine undergo deeper integration in the future, these techniques hold considerable promise for providing more precise guidance for NT efficacy evaluation and regimen optimization in oncology.

### Challenges and limitations of PET/CT technology

The overall efficacy of PET/CT in predicting the therapeutic response to neoadjuvant therapy in BC is relatively modest, and pertinent investigations remain sparse. Specifically, the diagnostic accuracy of conventional ¹⁸F-FDG PET/CT is significantly influenced by the molecular subtype of the tumor. Its predictive value for ALN status is limited, and in certain subtypes, a robust and consistent correlation between PET parameters and pCR is lacking. Within the domains of radiomics and ML, although the constructed predictive models exhibit promising potential, their advancement is currently constrained by insufficient sample sizes and a paucity of independent external validation, thereby compromising both model generalizability and clinical applicability. Conversely, novel molecular probes such as ⁶⁸Ga-FAPI PET/CT demonstrate superior sensitivity and specificity relative to FDG and are capable of furnishing earlier information pertaining to treatment response. Nevertheless, research in this area remains at a preliminary stage and has yet to undergo rigorous validation through large-scale prospective studies. In summary, for PET/CT technology to achieve clinical translation in the realm of individualized therapeutic response prediction, a series of pivotal challenges must be addressed, including those related to subtype heterogeneity, model reproducibility, and the standardization of novel probes.

## Emerging technologies

Beyond the imaging modalities currently employed in routine clinical practice for response assessment (such as US, MRI, CT, and PET/CT), a series of emerging imaging technologies have been developed and applied to the evaluation of NT efficacy in recent years, concomitant with rapid advancements in imaging science. Table 5 presents the applications of various novel imaging techniques in assessing neoadjuvant treatment response for breast cancer.

**Table 5:** Comparison of emerging imaging techniques for assessing neoadjuvant treatment response in breast cancer

Technique	Principle	Primary evaluation metrics	Advantages	Limitations	Clinical Application Scenarios	Refs
NIRF	Uses targeted fluorescent probes excited by NIR light, detecting emitted fluorescence for molecular imaging	Fluorescence intensity, Target-to-background ratio	Ultra-high sensitivity; Real-time imaging (e.g., intraoperative navigation); No ionizing radiation	Shallow tissue penetration (<1–2 cm); Most probes in preclinical stage; Requires dedicated equipment	Mainly for intraoperative tumor margin delineation, sentinel LN biopsy; Preclinical research for in vivo target tracking	^129^
DOBI	Uses NIR light to detect differences in Hb and HbO₂ concentration in breast tissue	BS, OS	Non-invasive, no compression, no radiation; Assesses tumor blood oxygen metabolic function	Limited depth information, low spatial resolution; Affected by tissue composition (e.g., fat thickness)	Auxiliary functional screening tool for monitoring hemodynamic changes in early efficacy	^137^
NIRS tomography	Uses NIR light to probe tissue optical properties, quantifies hemoglobin concentration and oxygen saturation	HbT, StO₂	Non-invasive, no radiation, bedside repeatable monitoring; Provides blood flow/oxygenation metabolism info	Limited penetration depth (<2–3 cm); Affected by probe pressure; Lack of standardization	Prospective research; Early, frequent monitoring of tumor blood flow and metabolic response	^139^
DOT	Uses NIR light scattering in tissue, reconstructs 3D distribution of internal optical parameters via inverse problem algorithms	HbT, StO₂, Water content	No radiation, wide-field imaging, provides quantitative blood oxygen metabolic parameters	Low spatial resolution; Complex image reconstruction algorithms; Results model-dependent	Continuous monitoring of blood oxygen metabolic dynamics pre/post-treatment for early response prediction	^31^
DCS/DOSI	DCS: Measures tissue blood flow index (BFI) via dynamic light scattering signals; DOSI: Measures HbT, StO₂, etc., can calculate oxygen metabolic rate (MRO₂)	BFI, StO₂, MRO₂	Unique ability for non-invasive, bedside quantitative measurement of tissue blood flow & MRO₂; Allows frequent monitoring	Limited detection depth; Motion-sensitive; Dedicated equipment, not yet commercially widespread	Cutting-edge research, precise monitoring of tumor hemodynamics and oxygen metabolism response to therapy, exploring resistance mechanisms	^32^

*Hb* Hemoglobin, *HbO₂* oxygenated hemoglobin, *BS* blood score, *OS* oxygenation score, *HbT* total hemoglobin concentration; *StO₂* tissue oxygen saturation, *BFI* blood flow index, *MRO₂* metabolic rate of oxygen.

### Near-infrared fluorescence (NIRF)

Biophotonic imaging overcomes the inherent limitations of conventional imaging modalities and holds considerable promise within the rapidly evolving domain of in vivo imaging. NIRF imaging is a biomedical imaging methodology predicated on optical principles. Its core mechanism involves the utilization of near-infrared (NIR) light at specific wavelengths to excite fluorescent probes. Upon binding or reacting with their intended targets, these probes undergo alterations in molecular structure, and the resultant fluorescent signals emitted upon excitation are detected to facilitate the localization and visualization of biological targets^129^. This technique represents a pivotal convergence of molecular and functional imaging. By virtue of its high sensitivity, capacity for real-time dynamic monitoring, and superior tissue penetration, NIRF imaging has demonstrated substantial clinical utility in areas such as oncological theranostics^130^, immunotherapy surveillance^131^, and intraoperative navigation^132^. While investigations have been conducted in malignancies including bladder cancer^133^ and pancreatic ductal adenocarcinoma^132^, research concerning its application in assessing NT efficacy for BC remains notably absent, thereby delineating a significant direction for future exploration within this context. Furthermore, despite its considerable potential, only a limited number of probes (e.g., ICG, Cytalux) have currently secured clinical approval^134–136^. The majority of nanoprobes remain confined to preclinical investigational stages, and their safety profiles, efficacy, and standardization protocols necessitate systematic validation.

### Optical imaging based on hemoglobin metabolism

Optical imaging techniques, by capturing biophysical alterations across distinct tissue compartments—encompassing the vascular, intracellular, and extracellular matrix domains—facilitate efficient and effective longitudinal monitoring as well as early prediction of therapeutic response. DOBI is a functional tissue imaging modality predicated on NIR spectroscopy. This technique achieves quantitative assessment of tumor blood score (BS) and oxygen score (OS) by detecting the differential and specific absorption of NIR light by various constituents within breast tissue, particularly hemoglobin (Hb), oxyhemoglobin (HbO₂), and deoxyhemoglobin (HHb)^21^. A prospective cohort study by Zhang J et al. revealed that BC patients within the chemotherapy-sensitive cohort exhibited a characteristic temporal trend one day prior to the second chemotherapy cycle, manifesting as a decrease in BS concomitant with an increase in OS. This observation suggests that these two parameters may serve as reliable functional imaging biomarkers for the early identification of individuals who are non-responsive to chemotherapy^137^.

NIRS tomographic technology enables the non-invasive surveillance of biophysical alterations occurring within the vascular, cellular, and extracellular matrix compartments of tumor tissue^138^. Such alterations provide a crucial foundation for the early assessment of NAC response. In a prospective cohort investigation, Cao X et al. conducted a systematic analysis of the dynamic change in total hemoglobin concentration (ΔHbT%) within the tumor region relative to the contralateral normal breast tissue. The findings demonstrated that ΔHbT% measured via NIRS tomography at the conclusion of the first chemotherapy cycle could predict RCB-0 and RCB-II with high precision, achieving AUC values of 0.97 and 0.94, respectively. Specifically, a decline in HbT concentration was indicative of a favorable therapeutic response (RCB-0), whereas an elevation in HbT signified the presence of residual disease (RCB-II/III)^139^.

### Optical imaging based on diffuse light and hemodynamics

DOT, an emerging functional optical imaging modality, enables non-invasive, label-free, wide-field imaging through the detection of diffusely propagated NIR light following tissue absorption and scattering. It facilitates the quantitative acquisition of concentration information pertaining to tissue hemoglobin oxygenation status (HbO₂/HHb), oxygen saturation (StO₂), water, lipid, and melanin content, thereby constituting a valuable tool for the functional assessment of tumors^140^. Zhu Q et al. innovatively integrated US with US-guided DOT, and their prospective investigation revealed that the combination of the percentage reduction in tumor diameter (%US) and total hemoglobin concentration (%HbT) after the first treatment cycle, together with HER2/ER receptor status, yielded the optimal performance for predicting pCR (AUC = 0.941; 95% CI 0.869–1.0)^141^. This finding is mutually corroborated by the work of Deng B et al.^31^, whose study additionally demonstrated that patients in the pCR cohort exhibited a sustained decline in HbT and a persistent negative alteration in StO₂ throughout the entire course of therapy, observations that are also concordant with the conclusions drawn by ref. ^139^. Collectively, these results indicate that DOT technology, by capturing functional modifications associated with early therapeutic response, may contribute to averting the cumulative toxicity and financial burden inherent to ineffective treatment.

DCS and DOSI, as nascent optical functional imaging techniques, are capable of furnishing functional information that transcends the scope of conventional anatomical imaging. Predicated upon the principles of NIR light interaction with biological tissues, these two modalities provide distinct non-invasive means for monitoring tumor response to NT. Specifically, DCS quantitatively assesses the tissue microvascular BFI through the detection of dynamic light scattering signals, thereby reflecting real-time tumoral hemodynamic alterations^142^. In contrast, DOSI non-invasively quantifies the concentrations of HbO₂, HHb, water, and lipid within tumor tissue via NIR spectroscopy. This not only facilitates calibration of DCS-derived data but also permits the subsequent calculation of the MMRO₂, thereby enabling a comprehensive evaluation of the tumor’s metabolic status^143^. Multiple investigations have substantiated a significant correlation between these optical parameters and pCR. For instance, the study by Cochran JM et al. demonstrated that at the mid-point of therapy, the rBFI₇/_n_ in patients achieving pCR approached a normalized level, whereas it remained elevated in non-pCR patients. This observation suggests that the disparity in BFI and rBFI₇/_n_ at the mid-treatment juncture may serve as a potential biomarker for the early prediction of pCR^144^. This investigation represents the inaugural application of DCS for the continuous monitoring of NAC-induced hemodynamic changes in human subjects.

**Fig. 4:**
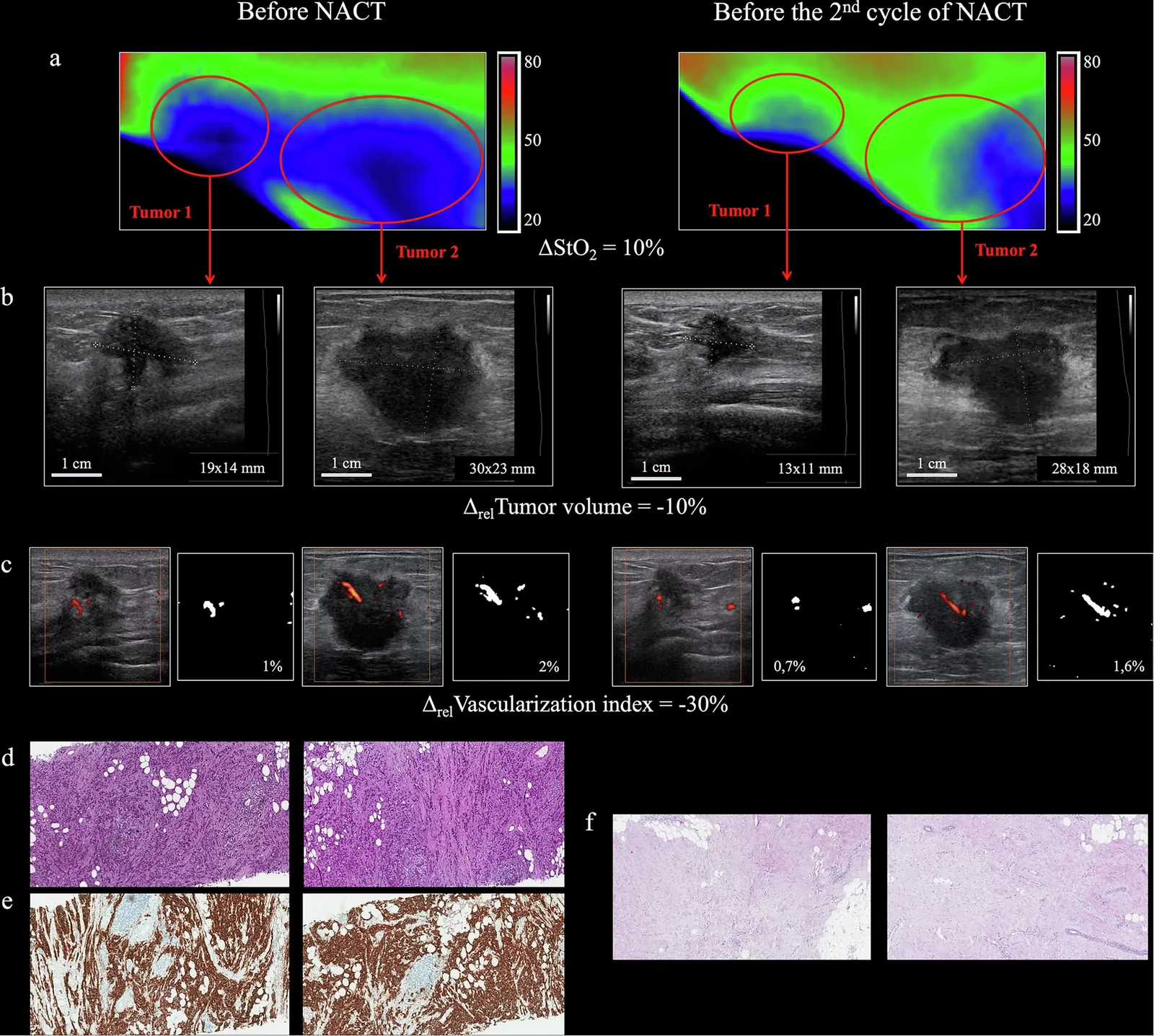
Changes in the tumor oxygen saturation, tumor vascularization, and tumor volume of patient B, aged 33. Diagnosis: multifocal (2 nodes) right BC, stage IIa (T2mN0M0), invasive breast carcinoma of no special type G3 (**d** × 100, pretreatment histopathology) HER2neu-positive cancer (estrogen receptors = 0 points, progesterone receptors = 0 points, Ki67 = 60% (**e** × 100, pretreatment immunohistochemistry)). After one cycle of NACT, DOSI showed an increase in the oxygenation level by 10% (**a**). US revealed a 10% decrease in tumor volume (**b**) as well as a 30% decrease in tumor vascularization (**c**). A pathologic investigation after six NACT cycles and surgery failed to find vital tumor cells, equivalent to the five grade of pathological tumor response (**f** × 100, postoperative histology showing pCR). Copyright 2023, Breast Cancer Res, CC BY 4.0.

Further investigation has revealed that disparate therapeutic regimens may exert influence upon the hemodynamic characteristics of the tumor through distinct biological mechanisms. A prospective, dual-center study by Tank A et al., employing DOSI technology, ascertained that the pattern of change in HbO₂ was highly contingent upon the specific therapeutic protocol (maximum tolerated dose versus metronomic chemotherapy). Notably, DOSI facilitated the prediction of therapeutic efficacy in the maximum tolerated dose cohort through alterations in HbO₂ observable as early as the first day of treatment^145^. This perspective is reinforced by the research of Pavlov MV et al., who identified alterations in tumor oxygenation as the most efficacious indicator for the early prediction of BC response to NAC^32^ (an illustrative schematic from this study is presented in Fig. 4). As detection methodologies that obviate the need for contrast agents or ionizing radiation, DCS and DOSI possess the unique advantage of enabling multiple, serial bedside monitoring assessments, thereby demonstrating considerable promise for application in therapeutic response evaluation.

### Limitations and future prospects of emerging technologies

Although the aforementioned optical and functional imaging modalities have demonstrated high sensitivity and considerable potential in the early assessment of neoadjuvant therapy response, their overall development remains confined to preclinical or early-phase clinical research stages. Presently, the limited sample sizes of available studies constrain the generalizability of established predictive models and the universality of their conclusions. Despite the capacity of various optical techniques—ranging from DOBI, NIRS, and DOT to DCS/DOSI—to non-invasively capture early chemotherapy-induced alterations in parameters such as HbT, StO₂, and collagen density at the bedside without ionizing radiation, these methodologies remain technically heterogeneous. They are collectively encumbered by challenges including sensitivity to probe positioning and applied pressure, a restricted field of view, and an absence of standardized protocols for image acquisition and quantitative parameter analysis, which collectively impede direct comparison and integration of findings across disparate studies. Notably, research concerning the application of techniques such as NIRF imaging in the assessment of NAC efficacy remains conspicuously absent. Future progress will hinge upon the advancement of large-scale, multicenter, prospective clinical validation, the establishment of standardized operating and data analysis protocols, and the exploration of multimodal integration strategies that combine different optical technologies or incorporate them with conventional anatomical imaging. These endeavors represent critical directions for overcoming current bottlenecks and ultimately enabling these non-invasive functional imaging techniques to inform individualized therapeutic decision-making in BC.

## Discussion and perspectives

**Fig. 5:**
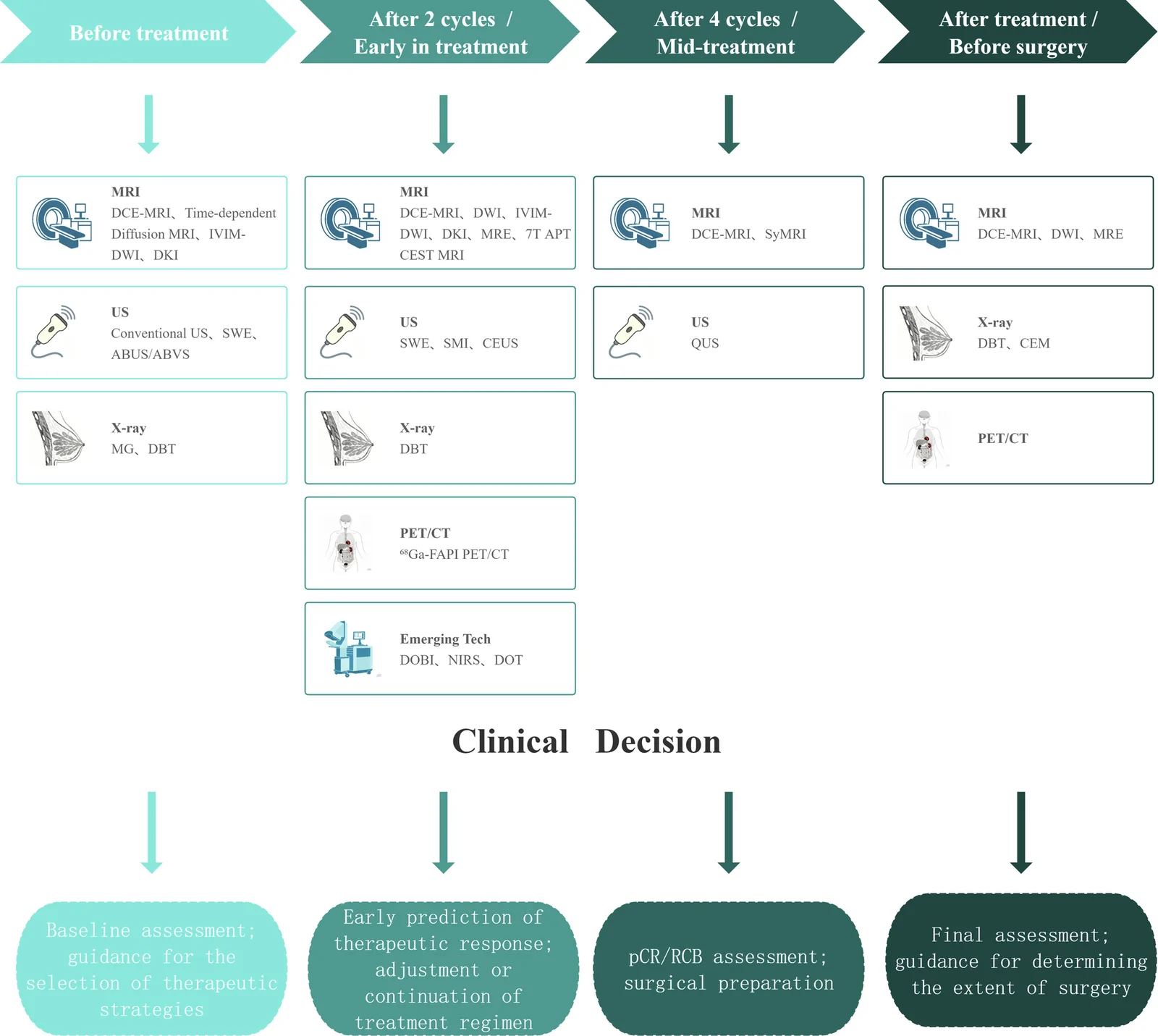
Clinical pathway diagram for imaging-based assessment at distinct timepoints during NT for BC.

This review has primarily compared the utility of US, X-ray-based imaging, MRI, PET/CT, emerging optical imaging modalities, and AI in the assessment of neoadjuvant therapy efficacy for patients with BC. Within the imaging strategies employed to evaluate NT response, distinct modalities possess unique advantages and correspondingly suitable application scenarios. Clinical implementation necessitates individualized selection predicated upon the specific circumstances of each patient. The applicable timepoints and specific contexts for various imaging examinations are comprehensively detailed in Table 6 and Fig. 5.

**Table 6:** Applicable timepoints and clinical scenarios for imaging examination techniques

Timepoint of action	Preferred molecular subtype	Recommended technique	Key indicators	Clinical action
Before treatment	All subtypes, particularly TNBC/HER2+	MRI (DCE-MRI, time-dependent diffusion MRI, IVIM-DWI, DKI)	Breast parenchymal background, cellular density, histogram parameters	Baseline assessment; prediction of the likelihood of achieving pCR; identification of resistant subtypes; guidance for the selection of therapeutic strategies (e.g., whether to intensify or de-escalate treatment)
	Luminal B subtype	US (conventional, elastography, ABUS/ABVS)	Peritumoral characteristics	
	Luminal B subtype/TNBC	X-ray (MG, DBT)	ΔVbd%, mass size, original tumor surface-to-volume ratio	
After two cycles/Early in treatment	TNBC/HER2+	MRI (MRI, DCE-MRI, DWI, IVIM-DWI, DKI, MRE, 7 T APT CEST MRI)	ALN volume, percentage reduction in tumor volume, ΔADC%, histogram parameters, PAR	Early prediction of therapeutic response; identification of non-responders to enable adjustment of treatment regimens (e.g., switching chemotherapeutic agents or proceeding to early surgery); avoidance of ineffective therapy
	Luminal B subtype	US (SWE, SMI, CEUS)	Blood flow velocity, RI	
	-	X-ray (DBT)	Mass size	
	HER2+	PET/CT (⁶⁸Ga-FAPI)	Semiquantitative parameters, HER3 protein expression	
	-	DOBI、NIRS、DOT	BS, OS, ΔHbT%	
After four cycles/Mid-treatment	-	US (QUS)	Autocovariance and Laws’ texture energy (SSLS)	Confirmation of therapeutic response trends; assessment of whether pCR or specific RCB class has been achieved; guidance for subsequent regimen planning or surgical preparation
	TNBC	MRI (DCE-MRI, SyMRI)	FTV, quantitative parameters	
After treatment/ Before surgery	All subtypes	MRI (MRI, DCE-MRI, DWI, MRE)	Mass size, ΔADC%, TSR	Final preoperative assessment; prediction of pCR; guidance for determining the extent of surgery (e.g., breast-conserving surgery, axillary lymph node dissection)
	Luminal B/ Luminal B + HER2+	PET/CT	Semiquantitative parameters	
	-	X-ray (DBT, CEM)	Mass size, calcifications	

*ALN* axillary lymph node, *ΔVbd%* change in volumetric breast density percentage, *ΔADC%* percent change of ADC, *PAR* phase angle ratio, *RI* resistance index, *BS* blood score, *OS* oxygenation score, *ΔHbT%* dynamic change rate of total hemoglobin concentration, *FTV* functional tumor volume, *TSR* tumor stiffness ratio.

MRI, by virtue of its multiparametric and multimodal imaging capabilities, excels in the evaluation of tumoral morphological characteristics, functional status, and microenvironmental alterations. It assumes a pivotal role across the various phases of NT in BC patients and is currently regarded as the most accurate imaging modality for assessing therapeutic response^146^. This technique is particularly indicated for patients requiring high-precision assessment of tumor infiltration extent, early response monitoring, or those harboring highly heterogeneous tumors. Nevertheless, the clinical application of MRI is encumbered by constraints including protracted examination duration, sensitivity to metallic implants, and comparatively elevated procedural costs. Concurrently, not all healthcare institutions are equipped with dedicated high-field-strength MRI systems specifically designed for breast imaging, nor do they uniformly possess radiologists with specialized expertise in acquisition and interpretation. Consequently, its utilization is predominantly concentrated within comprehensive hospitals. US, when augmented by techniques such as elastography and hemodynamic parameter assessment, offers distinct advantages including real-time capability, absence of ionizing radiation, reproducibility, and relative cost-effectiveness. It is well-suited for deployment in primary care settings and for patients necessitating frequent follow-up surveillance. US holds particular value in the initial evaluation of early therapeutic response trends and the preliminary assessment of ALN, thereby playing a critical role in guiding the formulation of axillary surgical strategies. X-ray-based techniques, leveraging their high sensitivity for detecting microcalcification clusters, are especially applicable for monitoring therapeutic response in BC patients whose disease is predominantly characterized by calcific manifestations. Furthermore, this modality can monitor alterations in mammographic density, thereby providing adjunctive information for response assessment. Owing to its expediency and favorable reproducibility, X-ray imaging is also amenable to widespread dissemination. PET/CT demonstrates notable utility in the assessment of systemic metabolic activity, the detection of distant metastatic disease, and the evaluation of therapeutic response within specific molecular subtypes (e.g., TNBC), rendering it appropriate for staging evaluations or for guiding response determination predicated on metabolic activity.

In the clinical decision-making process, imaging assessment should encompass a comprehensive consideration of multiple factors. From a health economics perspective, the relationship between examination frequency and cost-effectiveness must be judiciously balanced to avert excessive imaging. The practical challenge of limited access to advanced imaging equipment in primary healthcare institutions can be partially mitigated through algorithmic optimization; however, this necessitates a stratified management strategy. Specifically, primary care settings can leverage US technology as a foundational platform, augmented by auxiliary diagnosis employing US-based radiomics or DL models. Prior investigations have demonstrated that US radiomics models can achieve AUCs ranging from 0.845 to 0.931 for pCR prediction^59–61^, while their associated costs are merely one-fifth to one-tenth those of MRI. Regional medical centers may judiciously adopt non-contrast techniques (such as DWI and SyMRI) as alternatives to DCE-MRI, thereby reducing reliance on specialized hardware prerequisites. Within comprehensive hospitals, a workflow integrating multiparametric MRI with AI-assisted diagnosis can be comprehensively implemented. Regarding contrast agent utilization, a thorough evaluation of the patient’s hepatic and renal function, as well as any history of allergies, is imperative to prioritize the selection of examination protocols with superior safety profiles. For young patients or those with renal insufficiency who require repeated follow-up surveillance, preference should be accorded to imaging combinations that are devoid of ionizing radiation, obviate the need for contrast injection, or minimize contrast dependency (e.g., US combined with DWI and optical imaging), rather than the fixed application of DCE-MRI or PET/CT. The judicious selection of local or whole-body imaging modalities should be predicated upon the pathological subtype of the tumor and the presence or absence of distant metastasis. For patients exhibiting inconspicuous anatomical alterations post-treatment yet demonstrating functional or metabolic perturbations, adjunctive functional imaging modalities—including DWI, DCE-MRI, and PET—may be employed to enhance the discriminative capacity between residual disease and treatment-related changes. The aforementioned imaging-derived information can directly inform the delineation of surgical resection margins and guide the formulation of ALN management strategies (e.g., circumventing unnecessary ALN dissection). Furthermore, it facilitates the early identification of ineffective therapeutic strategies, thereby enabling timely adjustments to the treatment regimen and ultimately contributing to the attainment of de-escalation therapy and precision medicine objectives.

Despite the notable advancements achieved in the application of imaging techniques for the assessment of NT efficacy, numerous common challenges persist. First, the lack of standardization in imaging acquisition protocols and evaluative criteria across different institutions compromises the comparability of results. Addressing this issue necessitates the execution of large-scale, multicenter clinical investigations to establish unified operational standards or expert consensus, thereby minimizing subjective interpretive variance and enabling precise cross-study comparisons. Second, the effective translation of imaging-based predictions of non-pCR into concrete subsequent therapeutic modifications (e.g., altering chemotherapeutic regimens, incorporating targeted agents, or proceeding to early surgical intervention) currently lacks substantiation from prospective clinical trial evidence predicated upon imaging biomarker-driven strategies.

Third, the intrinsic heterogeneity of tumors, coupled with complex biological responses such as therapy-induced fibrosis and necrosis, further compounds the difficulty of image interpretation. This underscores an escalating imperative for the integration of functional or optical imaging modalities to facilitate more precise evaluation of dynamic biological alterations within both intratumoral and peritumoral territories. Such integration, encompassing morphological, functional, and molecular imaging data, is essential for comprehensively enhancing the precision of therapeutic response monitoring.

Fourth, the majority of current AI models rely on single-center retrospective data and lack prospective, multicenter validation, thereby incurring a risk of overfitting. Concurrently, the decision-making mechanisms of DL models remain opaque, with most AI investigations still operating in a “black box” paradigm, yielding only predictive probabilities without accompanying interpretability. This, to a certain extent, constrains the reliability of their clinical translation and application. In response to the aforementioned issues, researchers both domestically and internationally have proposed systematic solutions from multiple perspectives^147–150^.

With regard to data standardization and multicenter validation, Bi, W. L. et al. have noted that the absence of unified standards for the annotation, segmentation, and quality control of medical imaging data constitutes a major obstacle to the clinical translation of AI^147^. It is recommended that imaging acquisition parameters, region-of-interest delineation protocols, and standardized settings for feature extraction software be harmonized, with the mandatory allocation of an independent external validation cohort. The paradigms established by the I-SPY 2 trial^114^ or the ACRIN 6657 study^87^ may serve as instructive models in this context. Pettit, R. W. et al. have also emphasized that data preprocessing represents the most time-consuming phase in AI model development and must rigorously adhere to standardized workflows encompassing feature construction, outlier removal, missing value imputation, and dimensionality reduction to ensure model reliability and reproducibility^148^.

Addressing the issue of class imbalance, Abbasian Ardakani A et al. have indicated in their review that minority classes (e.g., patients achieving pCR) are typically the primary focus of clinical interest in medical data, and misclassification of these cases may incur substantially higher healthcare costs^149^. Consequently, it is recommended that metrics such as the Matthews correlation coefficient (MCC), F1 score, balanced accuracy, and the area under the precision-recall curve (AUPRC) be employed in lieu of conventional accuracy to more reliably evaluate model performance on imbalanced datasets.

Turning to the mitigation of overfitting and the risk of “cherry-picking” optimal models, Reyna, M. A. et al. have underscored that traditional evaluation metrics (e.g., AUC) lack clinical context, as they treat all correct diagnoses as equally important and all erroneous diagnoses as equally detrimental, thereby failing to reflect actual clinical needs^150^. It is therefore advisable to incorporate clinically contextualized evaluation metrics—for instance, assigning higher scores to accurate early predictions while imposing differential penalties for missed diagnoses and false alarms—to more faithfully represent the clinical utility of a model. Furthermore, independent external validation sets and K-fold cross-validation should be utilized to enhance model generalizability^149^.

Concerning the “black box” nature and interpretability challenges inherent to DL models, Abbasian Ardakani et al. have introduced Grad-CAM as a visualization tool capable of highlighting the critical image regions upon which the model focuses its attention, thereby assisting clinicians in understanding the rationale underlying model decisions^149^. Select studies (e.g., those by ref. ^62^ and ref. ^112^) have already begun to attend to model interpretability. Concurrently, it has been emphasized that models should provide uncertainty quantification for predicted probabilities to enhance transparency and clinical trustworthiness. Bi, W. L. et al. have also observed that the interpretability of AI systems remains an active area of investigation; although current research often prioritizes performance gains over interpretability, transparency and explainability are nonetheless essential for fostering clinical confidence^147^.

Furthermore, efforts should be directed towards constructing a standardized evaluation framework that integrates multimodal imaging and diverse imaging technologies. Future predictive models could incorporate information from disparate imaging modalities, clinicopathological characteristics, and radiomics parameters, leveraging a multimodal fusion architecture for integrated analysis. The intrinsic relationship between radiomics features and genomic alterations or the immune microenvironmental context remains comparatively underexplored, with only a limited number of investigations—primarily based on MRI—having ventured into this domain. This area represents a particularly promising avenue for future research.

## Conclusion

The present review elucidates that the early and precise prediction and monitoring of neoadjuvant therapy efficacy in BC can be achieved through the integration of morphological, functional, and molecular information derived from multimodal imaging, coupled with in-depth analysis facilitated by artificial intelligence, thereby optimizing clinical decision-making.

## Data Availability

No datasets were generated or analysed during the current study.
